# *Streptococcus pneumoniae* Strains Isolated From a Single Pediatric Patient Display Distinct Phenotypes

**DOI:** 10.3389/fcimb.2022.866259

**Published:** 2022-03-31

**Authors:** Hannah N. Agnew, Erin B. Brazel, Alexandra Tikhomirova, Mark van der Linden, Kimberley T. McLean, James C. Paton, Claudia Trappetti

**Affiliations:** ^1^Research Centre for Infectious Diseases, Department of Molecular and Biomedical Science, The University of Adelaide, Adelaide, SA Australia; ^2^German National Reference Center for Streptoccocci, University Hospital Rheinisch-Westfälische Technische Hochschule (RWTH) Aachen, Aachen, Germany

**Keywords:** *Streptococcus pneumoniae*, virulence, raffinose, clinical isolates, carbohydrate metabolism, meningitis, bacteremia

## Abstract

*Streptococcus pneumoniae* is the leading cause of bacterial paediatric meningitis after the neonatal period worldwide, but the bacterial factors and pathophysiology that drive pneumococcal meningitis are not fully understood. In this work, we have identified differences in raffinose utilization by *S. pneumoniae* isolates of identical serotype and sequence type from the blood and cerebrospinal fluid (CSF) of a single pediatric patient with meningitis. The blood isolate displayed defective raffinose metabolism, reduced transcription of the raffinose utilization pathway genes, and an inability to grow *in vitro* when raffinose was the sole carbon source. The fitness of these strains was then assessed using a murine intranasal infection model. Compared with the CSF isolate, mice infected with the blood isolate displayed higher bacterial numbers in the nose, but this strain was unable to invade the ears of infected mice. A premature stop codon was identified in the *aga* gene in the raffinose locus, suggesting that this protein likely displays impaired alpha-galactosidase activity. These closely related strains were assessed by Illumina sequencing, which did not identify any single nucleotide polymorphisms (SNPs) between the two strains. However, these wider genomic analyses identified the presence of an alternative alpha-galactosidase gene that appeared to display altered sequence coverage between the strains, which may account for the observed differences in raffinose metabolic capacity. Together, these studies support previous findings that raffinose utilization capacity contributes to disease progression, and provide insight into a possible alternative means by which perturbation of this pathway may influence the behavior of pneumococci in the host environment, particularly in meningitis.

## Introduction

*Streptococcus pneumoniae* (the pneumococcus) is the world’s foremost bacterial pathogen, causing approximately 1.2 million deaths each year with over 190 million infections (Lavelle and Ward, 2021). This Gram-positive bacterium can asymptomatically colonize the nasopharynx of humans at a very high rate of up to 95% of infants and 25% of adults (Trimble et al., 2020). However, in a number of these carriers, pneumococci can disseminate from this niche to deeper mucosal sites within the body, such as the middle ear, to cause otitis media or they can invade sterile sites such as the lungs, blood, and brain, leading to invasive pneumococcal diseases (IPDs), such as pneumonia, bacteremia, and meningitis, respectively. Of the IPDs, meningitis has a high case fatality rate with 30% of cases in higher-income countries resulting in fatality and lower-income countries having a case fatality rate of 50% (Brouwer et al., 2010). Meningitis is caused by inflammation of the brain and spinal cord following bacterial infection. *S. pneumoniae* can disperse from the nasopharynx during colonization into the bloodstream, resulting in bacteremia. The bacteria carried in the blood cross the endothelial cell layer of the blood-brain barrier to invade the brain by receptor-mediated transcytosis, in which the bacterium binds a specific receptor on endothelial cells, facilitating the translocation through the barrier (Iovino et al., 2016). Replication of the pneumococci results in high levels of inflammation that damages the brain. Complications include cerebrospinal fluid pleocytosis, cochlear damage, hydrocephalus, and cerebrovascular complications. If left untreated, meningitis can result in severe health problems, including hearing loss, brain damage and death (Mook-Kanamori et al., 2011).

*S. pneumoniae* is a highly diverse and genetically plastic species, with at least 100 capsular serotypes superimposed on at least 16,000 sequence types (STs) (https://pubmlst.org/), identified by multilocus sequence typing (MLST) (Enright and Spratt, 1998; Kim and Weiser, 1998). The core genome is composed of approximately 1500 genes, accounting for ~70% of the genome, with the remaining ~30% made up of accessory regions (ARs), leading to increased diversity between STs (Obert et al., 2006). Capsule switching experiments have displayed that both serotype and genetic background (i.e. ST) affect virulence (Kelly et al., 1994; Mcallister et al., 2011), but strain complexity has made it difficult to determine if there is a link between ST/serotype and predisposition to cause invasive rather than localized disease, and the mechanisms by which pneumococci switch from commensalism to cause localized or invasive disease remain poorly understood.

Previous studies undertaken in our laboratory have demonstrated that *S. pneumoniae* clinical isolates of the same serotype and ST displayed different virulence phenotypes in mice, that correlated with their site of isolation in humans. Intranasal (i.n.) challenge in mice with clinical isolates of serotype 3 ST180, ST232 and ST233 showed that blood isolates preferentially spread to the blood, whilst ear isolates spread to the ear in a significant proportion of mice (Trappetti et al., 2013). Further studies using clinical isolates of serotype 14 ST15 showed that ear isolates were able to spread to the ear, whilst the blood isolates spread to the lungs of mice (Trappetti et al., 2013; Amin et al., 2015). Subsequent studies uncovered single nucleotide polymorphisms (SNPs) in matched blood and ear isolates of serotype 3 ST180 and serotype 14 ST15, including in genes within the raffinose uptake and utilization pathway for both STs, *rafK* and *rafR*, respectively. These SNPs affected growth when raffinose was the sole carbon source and influenced the expression of the raffinose utilization genes *aga, rafG* and *rafK* (Minhas et al., 2019). Exchanging these mutations between the serotype 14 strains showed that these phenotypes were attributable to the SNPs, uncovering novel and clinically relevant molecular features that favour distinct lifestyles of pneumococcal disease.

We have now sought to investigate the bacterial drivers of meningitis, using clinically relevant serotype 15C *S. pneumoniae* isolates obtained at the same time from a single patient, provided to us by the German National Reference Center for Streptococci (Aachen). Pneumococci serotype 15C blood and CSF isolates (designated 60B and 60CSF, respectively) were isolated in 2015 from a child, aged 2, admitted to hospital with meningitis. We have performed *in vitro* and *in vivo* phenotypic characterization and genome comparisons of these clinical isolates and found differences in carbohydrate metabolism and gene expression *in vitro*, and distinct pathogenic phenotype differences between the isolates *in vivo.*

## Materials and Methods

### Bacterial Strains and Growth Conditions

The *S. pneumoniae* strains used in this study are serotype 15C clinical isolates 60B (SN69534) and 60CSF (SN69531) (isolated from blood and CSF, respectively), D39 (serotype 2 ST595), and Rx1 (unencapsulated). Cells were routinely grown on Columbia agar supplemented with 5% (vol/vol) horse blood (BA), with or without gentamicin (40 µg/mL), at 37°C in 5% CO_2_ overnight (Mclean et al., 2020). Growth assays were performed with pneumococci grown in a chemically defined medium (CDM) (Kloosterman et al., 2006) comprising SILAC RPMI 1640 Flex Media, no glucose, no phenol red (Sigma), supplemented with amino acids (**Table 1A**), vitamins (**Table 1B**), uracil (0.01 mg/mL), adenine (0.01 mg/mL), choline chloride (0.005 mg/mL) and catalase (10 U/mL), and either 0.5% glucose, galactose, lactose, raffinose or melibiose.

**Table 1:** Components for the CDM.

	Components	Concentration in final media (mg/mL)
**A) Amino acids**	Alanine	0.24
	Glutamine	0.39
	Asparagine	0.35
	Arginine	0.125
	Lysine	0.44
	Isoleucine	0.215
	Methionine	0.125
	Phenylalanine	0.275
	Serine	0.34
	Threonine	0.225
	Tryptophan	0.05
	Valine	0.325
	Glycine	0.175
	Histidine	0.15
	Leucine	0.475
	Proline	0.675
	Cysteine hydrochloride	0.3
	Aspartic acid	0.3
**B) Vitamins**	Pyridoxal HCl	0.0028
** **	Thiamine	0.0014
** **	Pantothenic acid (Ca)	0.0014
** **	Biotin	0.00014
** **	Niacinamide	0.0014
	Riboflavin	0.0014
** **	Folic acid	0.0014

### MLST

To conduct MLST analysis, the *aroE, gdh, gki, recP, spi, xpt* and *ddl* genes of the blood strains were PCR amplified, purified using the QiaQuick Purification Kit (Qiagen), and sequenced as described previously (Enright and Spratt, 1998). Sanger sequencing was conducted by the Australian Genome Research Facility (AGRF). The sequence types (ST) of the pneumococci strains were determined by searching the MLST database (https://pubmlst.org/) for matching allelic profiles.

### Growth Assays

Strains were grown in flat-bottom 96-well microtiter plates (Costar) with a final volume of 200 µL as previously described (Minhas et al., 2019). Strains were inoculated at a starting optical density at 600 nm (OD_600_) of 0.05 in CDM supplemented with either 0.5% glucose, galactose, lactose, raffinose or melibiose, before being incubated at 37°C with 5% CO_2_. The OD_600_ was measured every 30 min for a total of 24 h in a SpectroSTAR Omega spectrophotometer (BMG Labtech). Assays were conducted in triplicate with at least three repeated independent experiments.

### Phenotypic Microarrays

Carbon phenotype microarray (PM) analysis using the PM microplates PM1 and PM2a (Biolog Inc.) was performed on the strains to test for the catabolism of 190 different carbon sources as previously described (Minhas et al., 2019). Every well of the microarrays contained a different carbon source. Briefly, cells were inoculated to a final OD_590_ of 0.06 in the buffer provided, according to the manufacturer’s instructions. This suspension was added in 100 µL aliquots to the wells, and the plate was incubated at 37°C, 5% CO_2_. The OD_590_ was measured every 15 min for 24 h. Catabolic activity was measured through colorimetric analysis. During catabolism, NADH is produced which subsequently reduces a colorless tetrazolium dye, resulting in a color change. The level of metabolism for each carbon source was arbitrarily determined by comparison with the negative value.

### Biofilm Assays

The formation of biofilms was measured in real time using the real time cell analyzer (RTCA) xCELLigence (Agilent Technologies Inc.) instrument, as described previously for *Streptococcus mutans* and *Staphylococcus* spp. (Gutierrez et al., 2016). This instrument detects variation in the impedance signal (expressed as the arbitrary cell index, CI) as bacterial cells attach and form biofilms on the gold-microelectrodes present in the bottom of the E-plates (Agilent Technologies Inc.) Briefly, strains were grown overnight on BA plates at 37°C with 5% CO_2_. Cells were harvested and resuspended in 200 µL of CDM + 0.5% glucose or galactose a final OD_600_ of 0.2. To the wells of the E-plate, 150 µL CDM + 0.5% glucose or 0.5% galactose was added before placing the plate in the cradle of the RTCA-DP system, within a 37°C incubator without CO_2_ supplementation. An initial baseline impedance reading was taken before the E-plates were removed and 50 µL of bacterial suspension was added to wells for a 1 in 4 dilution, bringing the starting OD_600_ to 0.05. For control wells, an additional 50 µL of CDM + 0.5% glucose or 0.5% galactose was added. E-plates were locked into the cradles of the RTCA-DP platform within the incubator and biofilm formation was monitored for 24 h by recording the impedance signal (CI) every 15 min. Assays were conducted in triplicate with at least two repeated independent experiments. Statistical analysis was performed using two-tailed Student’s *t* test; *P* values < 0.05 were deemed statistically significant.

### Adherence Assays

Adherence assays were carried out on the Detroit 562 human pharyngeal cell line as previously described (Trappetti et al., 2011). Briefly, cells were grown in Dulbecco’s modified Eagle’s medium (DMEM) supplemented with 10% fetal calf serum (FCS), 100 U/mL penicillin and 100 µg/mL streptomycin at 37°C in 5% CO_2_. Wells of 24-well tissue culture trays were seeded with 2 × 10^5^ Detroit cells in DMEM with 10% FCS and grown for 24 h before inoculation with pneumococci. These strains were grown overnight on BA plates, before being resuspended in CDM + 0.5% glucose or CDM + 0.5% galactose at a final OD_600_ of 0.2. 500 µL of each bacterial suspension was added to the washed Detroit cell monolayers. As a control, each bacterial suspension was added in the same volume to empty wells. After incubation for 2 h at 37°C, wells were washed 3 times with PBS before cells were detached from the plate by treatment with 100 µL 0.25% trypsin-0.02% EDTA and 400 μL of 0.1% Triton X-100 (Sigma). Samples were plated on BA to determine the number of adherent bacteria. Assays were conducted in triplicate with at least two repeated independent experiments. Statistical analysis was performed using two-tailed Student’s *t* test; *P* values < 0.05 were deemed statistically significant. Data are presented as percentage of adherent bacteria, calculated using the colony forming units per mL (CFU/mL) of adherent bacteria and the CFU/mL of bacteria from the control wells that underwent the 2 h incubation. Control wells were also used to monitor the growth of bacteria during the incubation time, ensuring that each strain had similar rates of growth.

### Capsule Assay

A FITC-dextran exclusion assay was used to determine the capsule thickness of strains based on previous methods (Hathaway et al., 2012), using FITC-Dextran 2000 kDa (Sigma). Briefly, pneumococci were grown overnight on BA plates in 37°C at 5% CO_2_. Cells were washed in PBS, before being resuspended to a final OD_600_ of 0.6 in 1 mL. A volume of 80 µL bacterial suspension was mixed with 20 µL FITC-dextran (10 mg/ml in MilliQ water). Each bacterial suspension was pipetted at a volume of 10 µL onto a microscope slide and a coverslip applied securely. Imaging was conducted on 3 separate days with freshly prepared bacterial suspensions each time. The slides were viewed and imaged using an Olympus FV3000 Laser Scanning microscope with a 60× objective (Adelaide Microscopy). The images were analyzed using Zeiss Zen imaging software. Statistical analysis was performed using the Kruskal-Wallis test; *P* values < 0.05 were deemed statistically significant.

### Targeted Gene Sequencing (Sanger Sequencing)

Selected genes of the raffinose operon from the strains underwent Sanger sequencing through AGRF (Adelaide, Australia). Briefly, samples were prepared for sequencing by PCR amplification of the region of interest using the primers listed in **Table 2**. PCR products were analyzed by gel electrophoresis, and successfully amplified products were purified using the QIAquick PCR Purification Kit (Qiagen) as per the manufacturer’s instructions. Purified PCR products were evaluated for DNA quantity and quality using a Nanodrop (Thermofisher). Samples were diluted in ultrapure nuclease-free water to a final concentration of either 18 ng/µL or 60 ng/µL depending on the size of the product, according to the Australian Genome Research Facility (AGRF) guidelines.

**Table 2:** Oligonucleotide primers used in this study for PCRs.

OLIGO NAME	SEQUENCE (5’ → 3’)	Reference
*seq_rafK_F*	AGAATCCAGTCAAATGTAGTGGAG	This study
*seq_rafK_R*	AAGTCAGTAATCATACGTACGGC	This study
*rafK_F*	AACGACGTAGCTCCAAAAGA	Minhas et al., 2019
*rafK_R*	GCTGGTTTACGTTCCAAGAA	Minhas et al., 2019
*seq_rafR_F*	TGTTTCAAAAGTAAGTAGCCATTTCG	This study
*seq_rafR_R*	TTCTTCTAGAATCTCTGGAAGAATAAGG	This study
*seq_rafEFG_F1*	GGCGAAGTTTACTCAGGTGC	This study
*seq_rafEFG_R1*	GCTGAACTCTTCCTCTGTGTC	This study
*seq_rafEFG_F2*	CACCATTTGGAATTGCAGGTG	This study
*seq_rafEFG_F3*	GAAAGCGGATGTGGATTAGGAG	This study
*seq_rafEFG_F4*	GCCTTTGACCAAGTCTTTGC	This study
*seq_rafEFG_F5*	ATTCCAGAAAGTCTGGATGAAGC	This study
*seq_rafK_F2*	TCAAATGTAGTGGAGAATCAGCG	This study
*seq_rafK_R2*	CGAAGAGTATTAAAGAGCATCACAATATAG	This study
*aga_F1*	TTCTATTTTGGAAAGCGATTTCAGG	This study
*aga_F2*	CTTTAGTGACTCATTCAGATCAGGG	This study
*aga_F3*	CCGCAATATCACTAAGCTAGGG	This study
*aga_F4*	GAAGCAGCTGTACAATTTAATTACGG	This study
*rafF_R*	AGAAGGTTTGGCCTTTGATT	This study
*aga_F_RT*	GTCAGACTAAGTTGAGCCTTAG	Minhas et al., 2019
*aga_R_RT*	CCAACTATACAGGTTCAGCA	Minhas et al., 2019
*rafG_F*	CCTATGGCAGCCTACTCCATC	Minhas et al., 2019
*rafG_R*	GGGTCTGTGGAATCGCATAGG	Minhas et al., 2019
*rafR_F*	CCAGCCATTCGTGATACATA	This study
*rafR_R*	CCTCCAGTGATTCCTAACCA	This study
*gyrA* RT F	ACTGGTATCGCGGTTGGGAT	Mclean et al., 2020
*gyrA* RT R	ACCTGATTTCCCCATGCAA	Mclean et al., 2020

### Genome Sequencing

*S. pneumoniae* strains were grown to mid-exponential phase in HI broth. Genomic DNA (gDNA) was extracted using the Promega Wizard^®^ Genomic DNA Purification Kit according to the manufacturer’s instructions, except the DNA pellet was rehydrated in ultrapure nuclease-free water. The lytic enzymes used were lysosome (30 mg/mL), 10 units mutanolysin and 0.1% sodium deoxycholate (DOC). The gDNA was sequenced at the South Australian Genomics Centre (South Australian Health & Medical Research Institute, Adelaide, South Australia) on an Illumina MiSeq (250-bp paired-end reads). Genome assemblies were generated using shovill v1.1.0 (Seemann, 2020a), a bacterial isolate *de novo* genome assembly pipeline based on SPAdes (Prjibelski et al., 2020). The resulting genome assemblies were annotated using Prokka v1.14.6 (Seemann, 2014). Variant analysis was conducted using Snippy v4.6.0 (Seemann, 2020b) by performing all pairwise combination of aligning reads from one sample to the genome assembly of another sample. Gene presence/absence analysis was determined with a reciprocal-best-hit approach applied to the Prokka-derived amino acid sequences using mmseqs2 v13.45111 (Steinegger and Söding, 2017). A 3-step iterative searching strategy was applied using the easy-rbh workflow of mmseqs2 using sensitivities of 1, 3 and 7 (command line arguments: –start-sens 1 –sens-steps 3 -s 7) using a k-mer length of 6 (command line argument: -k 6).

### RNA Extraction and qRT-PCR

Strains were initially grown overnight on BA plates at 37°C with 5% CO_2_. Cells were harvested, washed, and resuspended in 1 mL of CDM + 0.5% glucose or raffinose to a final OD_600_ of 0.2. Suspensions were incubated at 37°C with 5% CO_2_ for 30 min. RNA was extracted using a Qiagen RNeasy Minikit as per the manufacturer’s instructions. Differences in levels of gene expression were determined using one-step relative real-time qRT-PCR in a Roche LC480 real-time cycler, as previously described (Minhas et al., 2019). The specific primers used for the different genes are listed in **Table 2** and were used at a final concentration of 200 nM per reaction. Primers specific for *gyrA* mRNA were used an internal control. Amplification data were analyzed using the comparative critical threshold (2^-∆∆CT^) method (Livak and Schmittgen, 2001). Assays were performed in triplicate with a minimum of two independent experiments. Statistical analyses were performed using two-tailed Student’s *t* test; *P* values < 0.05 were defined as statistically significant.

### Murine Infection Model

Animal experiments were approved by the University of Adelaide Animal Ethics Committee. Female outbred 4- to 6-week-old CD-1 (Swiss) mice were anaesthetized by intraperitoneal injection of ketamine (8 mg/mL) and xylazine (0.8 mg/mL), and were challenged intranasally with 50 µL of bacterial suspension containing 1× 10^8^ CFU in SB as previously described (Minhas et al., 2019). The challenge dose was retrospectively confirmed by serial dilution and plating on BA. At 24 h and 48 h, groups of 6 or 8 mice were euthanized by CO_2_ asphyxiation before harvesting the blood, lungs, nasal tissue, ears, and brain. Tissues were homogenized and pneumococci enumerated as previously described by serial dilution and plating on BA plates containing 5 µg/mL gentamicin (Trappetti et al., 2011). Statistical analyses of log-transformed CFU data were performed using two-tailed Student’s *t* test; *P* values < 0.05 were deemed statistically significant.

## Results

### Initial Characterization of Serotype 15C Blood and CSF Clinical Isolates

We began characterization of serotype 15C blood and CSF isolates from the same patient, denoted 60B and 60CSF, by performing multi-locus sequence typing (MLST) to determine the sequence type (ST). Both 60B and 60CSF have the sequence type 8711 (ST8711). Additional information provided by the German National Reference Center for Streptococci included the antibiotic sensitivity of the isolates. Both isolates, 60B and 60CSF had the same MIC values for all the antibiotics tested, with all MICs falling below the clinical breakpoint (European Committee on Antimicrobial Susceptibility Testing; EUCAST), indicating full sensitivity for both the isolates (data not shown).

### Blood and CSF Isolates Metabolize Carbohydrate Sources Differently

**Figure 1:**
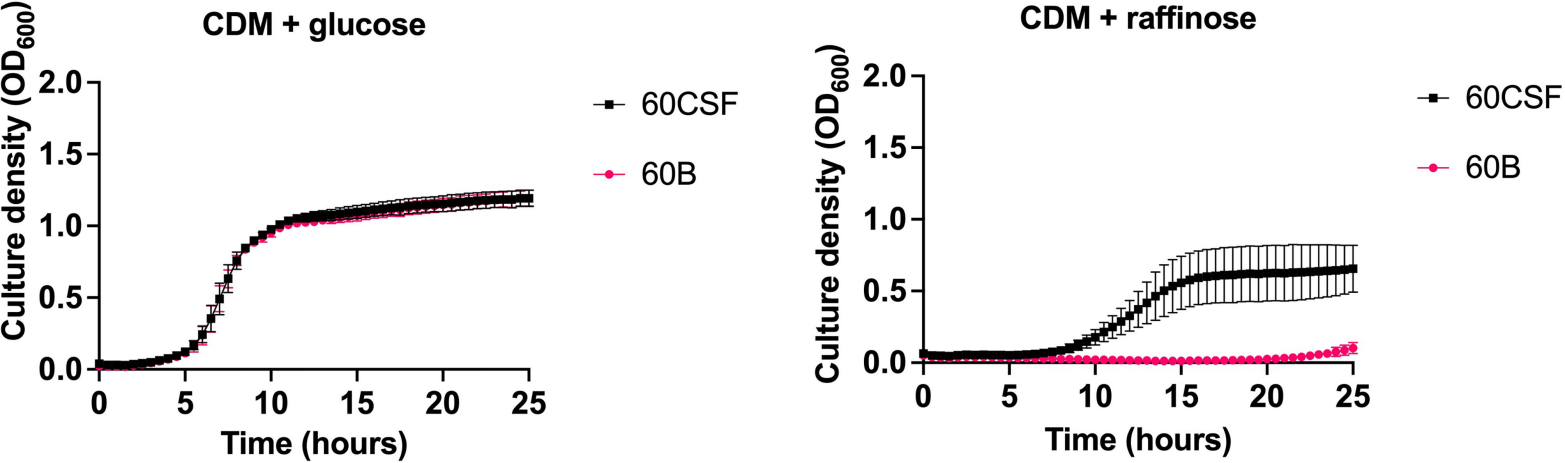
Growth of 60B and 60CSF in CDM + glucose and CDM + raffinose. *S. pneumoniae* serotype 15C ST8711 blood isolate (60B) and CSF isolate (60CSF) were grown in 200 μL CDM supplemented with 0.5% glucose (CDM + glucose) or 0.5% raffinose (CDM + raffinose). OD_600_ was measured every 30 min for 24 h. Data are mean OD_600_ ± standard error mean (SEM) from three independent assays, each performed in triplicate.

As previous research undertaken in our lab has shown that isolates of the same serotype/ST potentially metabolize the carbohydrate raffinose differentially, we investigated the carbohydrate metabolism capabilities of 60B and 60CSF. Two types of phenotypic microarray plates, PM1 and PM2a, with a total of 190 carbon sources were used to test the isolates’ metabolic ability. A total of 29 of the carbon sources are metabolized by both 60B and 60CSF, 10 carbon sources were metabolized by just 60CSF and the remaining 151 carbon sources were unable to be metabolized (**Table 3**). In line with prior research implicating an role for raffinose metabolism in tissue tropism, raffinose, and one of its derivatives, melibiose, were metabolized by 60CSF but not 60B. Of the carbon sources that were differentially metabolized by 60B and 60CSF, we analysed the growth of these strains in chemically defined medium (CDM) with the clinically relevant sugars: raffinose, melibiose, lactose and galactose. The growth of 60B and 60CSF were comparable in CDM + glucose (**Figure 1**), and the growth pattern was consistent in CDM + galactose and CDM + lactose (data not shown). Altought the phenotypic microarray showed that 60CSF was able to metabolize melibiose, there was no growth for either strain in CDM + melibiose, implying that melibiose may not be able to support growth of pneumococci as the sole carbon source in the present conditions (data not shown). Despite this, 60CSF was able to grow in CDM + raffinose, whilst 60B displayed an inability to grow in this medium (**Figure 1**). This indicates that despite the strains coming from the same patient and being closely related, there is a marked difference in their capacity to utilize raffinose as a carbon source.

**Table 3:** Phenotype microarray results for 60B and 60CSF in Biolog PM1 and PM2a plates.

CARBON SOURCE	60B	60CSF
L-Arabinose	+	+
N-Acetyl-D-Glucosamine	+	+
D-Galactose	+	+
D-Trehalose	+	+
D-Mannose	+	+
Glycerol	+	+
D-Xylose	+	+
D-Ribose	+	+
**L-Rhamnose**	**-**	**+**
D-Fructose	+	+
α-D-Glucose	+	+
Maltose	+	+
**D-Melibiose**	**-**	**+**
α-D-Lactose	+	+
Lactulose	+	+
Sucrose	+	+
ß-Methyl-D- Glucoside	+	+
Maltotriose	+	+
D-Cellobiose	+	+
N-Acetyl-ß-D-Mannosamine	+	+
D-Psicose	+	+
L-Lyxose	+	+
**Chondroitin sulfate c**	**-**	**+**
Dextrin	+	+
Inulin	+	+
Pectin	+	+
N-Acetyl-D Galactosamine	+	+
N-Acetyl Neuraminic Acid	+	+
**D-Arabinose**	**-**	**+**
**2-Deoxy-DRibose**	**-**	**+**
**3-0-B-D-Galactopyranosyl-DArabinose**	**-**	**+**
Palatinose	+	+
**D-Raffinose**	**-**	**+**
Salicin	+	+
**D-Tagatose**	**-**	**+**
**Turanose**	**-**	**+**
D-Glucosamine	+	+
5-Keto-DGluconic Acid	+	+
**Dihydroxy Acetone**	**-**	**+**

Catabolism was measured through a colorless tetrazolium dye being reduced by NADH produced during catabolism. +, metabolism occurred; -, no metabolism occurred. Metabolism was determined by calculating the change in OD_590_ from the initial to final measurements. These values were then compared with the change in the negative control and an arbitrary value based on the change in negative control was used to determine if metabolism occurred. Carbon sources which neither strain metabolized are not shown. Bold font indicates carbon sources that were differentially metabolized by the strains.

### Targeted Sequencing of the Raffinose Operon Reveals That *aga* Has a Premature Stop Codon in Both Isolates

**Figure 2:**
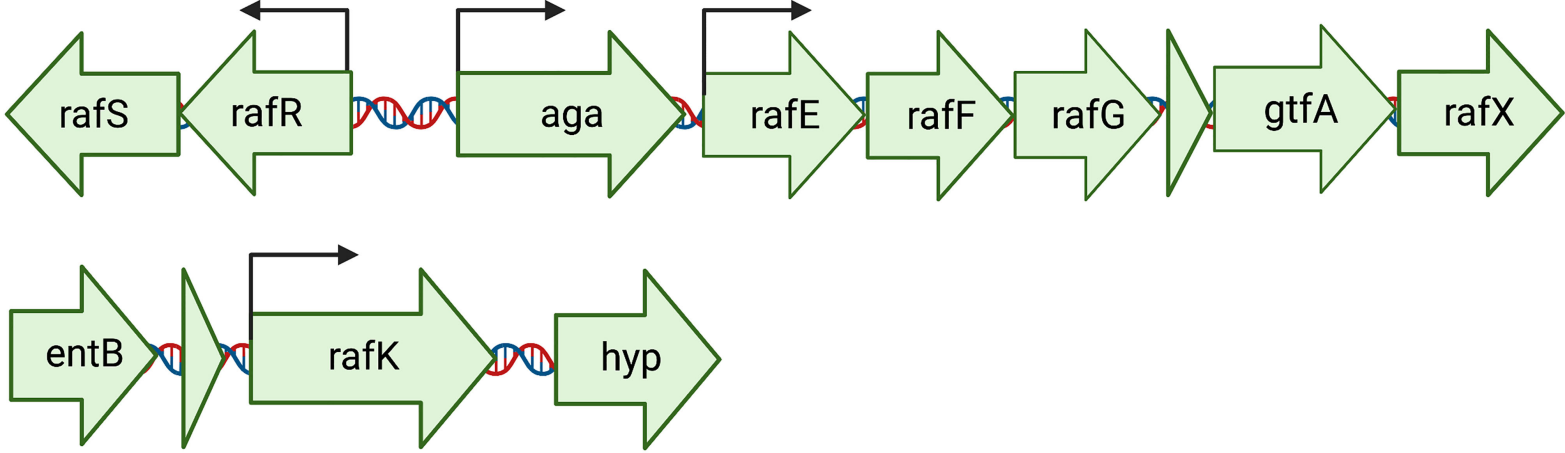
Arrangement of the genetic loci required for the import and utilization of raffinose by *S. pneumoniae*. The main locus contains 8 genes: an ABC transporter with solute-binding protein and two cognate permeases (*rafE*, *rafF* and *rafG*); two transcriptional regulators, *rafS* (repressor) and *rafR* (activator), which control the expression of the gene encoding an α-galactosidase (*aga*); a sucrose phosphorylase (*gtfA*), and *rafX*, encoding a gene of unknown function. Further upstream is *rafK*, encoding an ATP-binding protein, essential for raffinose import into the cell (Rosenow et al., 1999). Arrows above the genes indicate locations of promoters. Created with BioRender.com.

**Figure 3:**
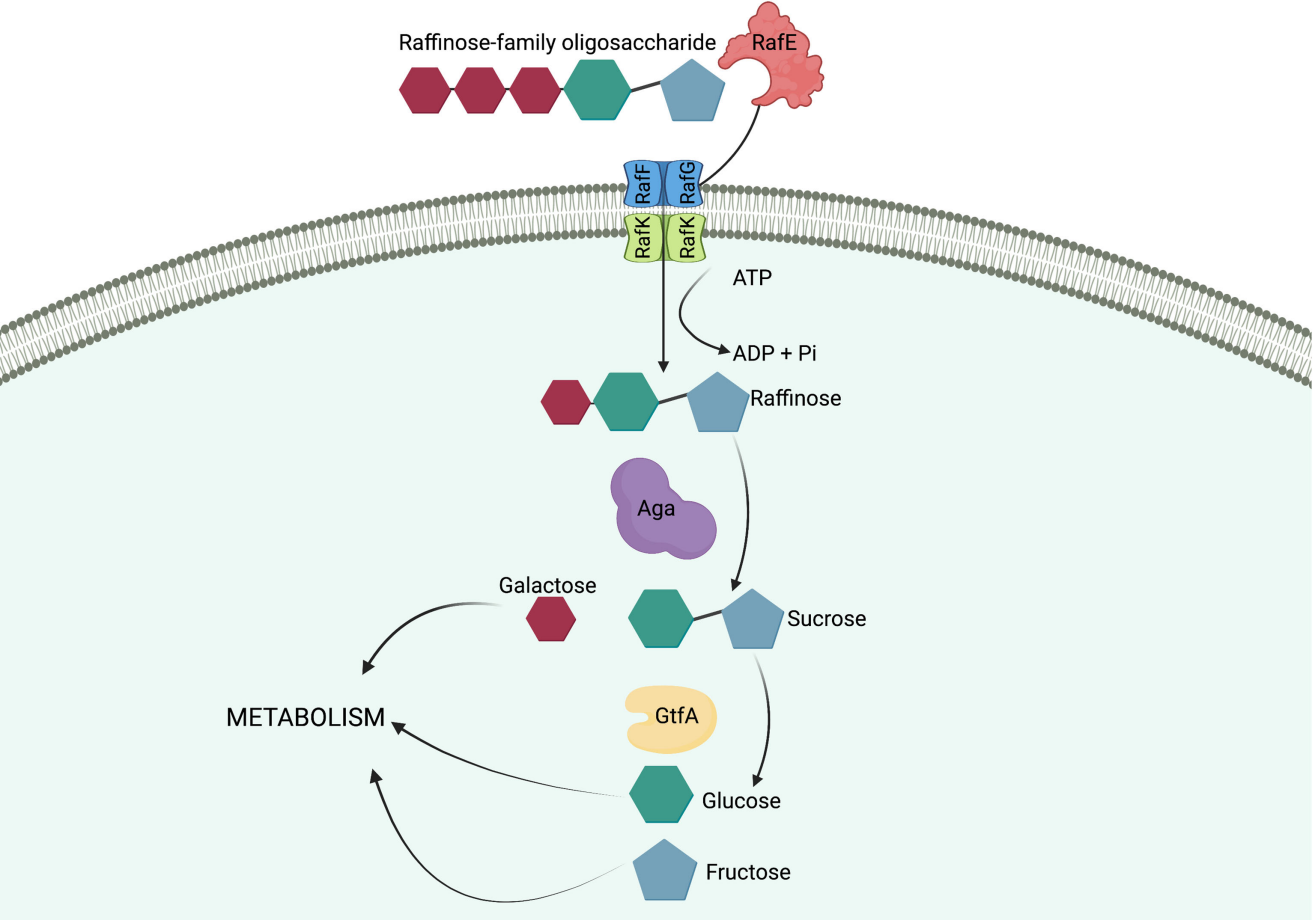
Import and breakdown of raffinose in *S. pneumoniae.* A raffinose-family oligosaccharide (RFO) is delivered to the ABC transporter cell membrane components, RafF and RafG, by the solute-binding protein, RafE. The RFO is imported into the cell using the energy produced from the ATP-binding protein, RafK, with concomitant ATP hydrolysis. In the cytoplasm of the cell raffinose is sequentially degalactosylated by an α-galactosidase, Aga, releasing galactose, which can enter other metabolic pathways, and sucrose. A sucrose phosphorylase, GtfA, cleaves sucrose into glucose and fructose, both of which can enter further metabolic pathways and be utilized by the cell. (Hobbs et al., 2019). Created with BioRender.com.

Considering that raffinose was only metabolized by and capable of supporting the growth of 60CSF, and not 60B, we performed Sanger sequencing targeting the genes, that are part of the core genome, that are critical for raffinose metabolism to investigate whether they were functional in both strains (**Figure 2**). The raffinose uptake and utilization operon consists of eight main genes encoding an α-galactosidase (*aga*), transcriptional regulators (*rafS* and *rafR*), an ABC transporter with solute-binding protein and two cognate permeases (*rafE*, *rafF* and *rafG*), a sucrose phosphorylase (*gtfA*), and a protein of unknown function (*rafX*). Additionally, further upstream there is a gene encoding an ATP-binding protein (*rafK*), which is essential for import of raffinose into the bacteria (Rosenow et al., 1999) (**Figure 2**). The *aga, rafR, rafE, rafF, rafG* and *rafK* were sequenced. Each of these genes encode proteins with a critical role in raffinose uptake and utilization (**Figure 3**). The sequences of all the genes investigated were the identical between the 60B and 60CSF strains. However, for both 60B and 60CSF, there was a SNP in *aga* relative to the published D39 and TIGR4 genome sequences that resulted in an early stop codon, truncating the Aga protein at 213 a.a., compared with 721 a.a. for native Aga (**Supplementary Figure 1**). As the gene sequences involved in raffinose uptake and metabolism were the same between 60B and 60CSF, we investigated if the expression of these genes correlated with the capacity to utilize raffinose by performing quantitative real-time reverse transcription-PCR (qRT-PCR).

### The Blood and CSF Isolates Display Differences in the Transcription of Raffinose Utilization Genes

**Figure 4:**
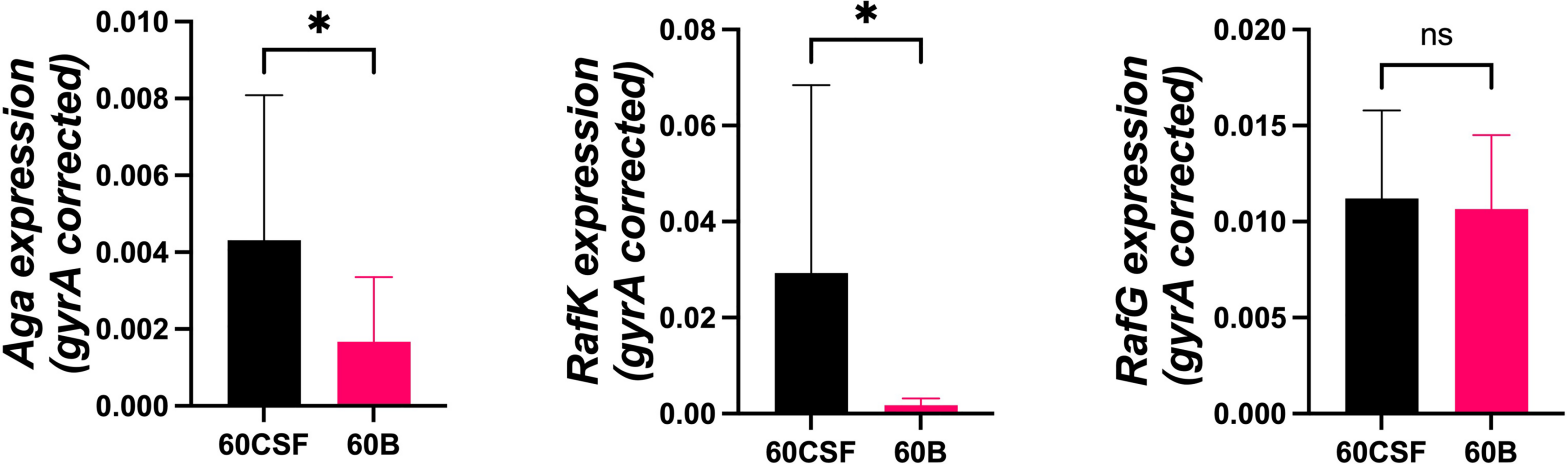
Expression of RafR-regulated raffinose pathway genes by 60B and 60CSF isolates. Strains were grown to OD_600_ 0.2 in CDM + glucose and then washed and resuspended in CDM + raffinose and incubated at 37°C for a further 30 min. RNA was then extracted, and levels of *aga*, *rafK*, and *rafG* mRNA were analyzed by qRT-PCR using *gyrA* rRNA as an internal control (see *Materials and Methods*). Data are mean OD_600_ ± standard deviation (SD) from two independent assays. Significance of differences in gene expression between isolates was determined using two-tailed Student’s *t* test; *P < 0.05. ns, not significant.

To determine if the difference in ability to utilize raffinose between the blood and CSF isolates corresponded with raffinose operon gene expression, 60B and 60CSF strains were grown to the same OD_600_ (0.2) in CDM + Glucose and then washed and resuspended in CDM + Raffinose and incubated for a further 30 min. RNA was then extracted, and levels of *rafR, aga*, *rafK*, and *rafG* mRNA, representative of each of the three RafR-regulated transcriptional units, were then measured relative to *gyrA* mRNA by qRT-PCR. Although *rafR* was not expressed at detectable levels in either strain (data not shown), the expression levels of the α-galactosidase (*aga*) and the ATP binding protein component of the transporter (*rafK*) were significantly up-regulated in the CSF isolate compared to the blood isolate (*P* < 0.05), while the expression level of the putative permease (*rafG*) was comparable in both strains (**Figure 4**). These results indicate that, while the component of the raffinose ABC transporter gene (*rafG*) was equally expressed in both strains, the uptake of raffinose may be limited by the reduced transcription of the gene encoding the ATP binding protein *rafK* required to energize this transporter.

### The Blood and CSF Isolates Display Altered Tissue Tropism

**Figure 5:**
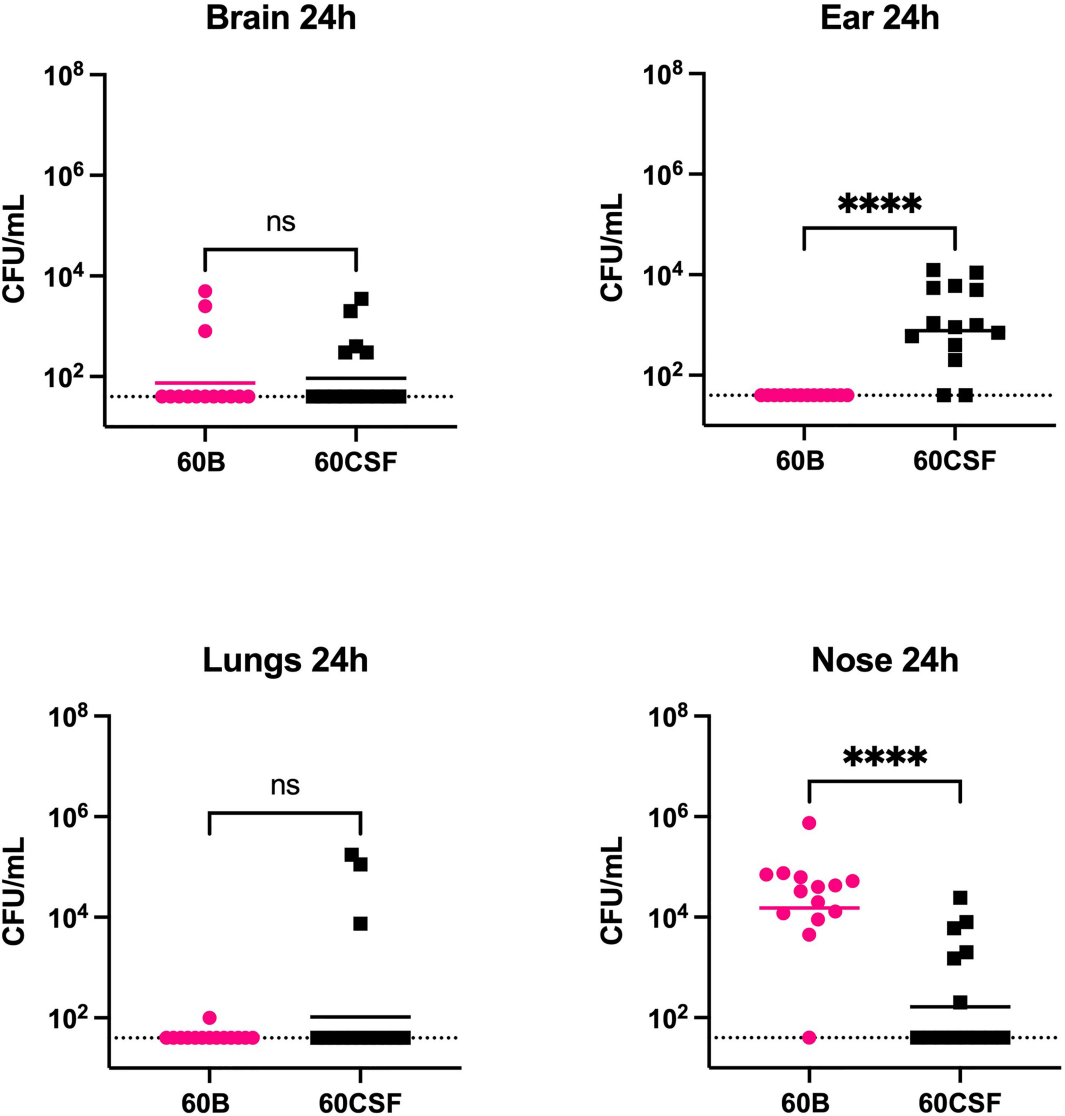
Virulence phenotypes of blood and CSF isolates. In 2 separate experiments groups of 6 or 8 mice were infected intranasally with 10^8^ CFU of the indicated strain. At 24 h, mice from each group were humanely euthanized and pneumococci in the brain, ear, lungs and nasal tissue were enumerated. Viable bacterial counts are displayed for each mouse in each niche; horizontal bars indicate the geometric mean (GM) CFU for each group; the dotted line indicates the detection threshold. Significance of differences in bacterial load between groups was determined using two-tailed Student’s t test; ****P < 0.0001. ns, not significant.

We next explored whether the two clinical isolates displayed any differences in disease phenotype using a mouse model of infection. Mice were intranasally challenged with 10^8^ CFU of each strain and the bacterial burden in the blood, brain, ears, lungs, and nose were assessed at 24 and 48 h post challenge. Mice challenged with either strain showed no detectable bacteria present in the blood at either time point (data not shown). At 24 h post challenge, there was no significant difference in the relative bacterial burden between the mice infected with the 60B or 60CSF isolates in the brain or lungs (**Figure 5**). No bacteria for the 60B isolate were detected in the ears at 24 h post challenge (**Figure 5**), while bacteria were present in the ears of over 85% of the mice for the 60CSF strain (**Figure 5**; *P* < 0.0001). Conversely, the bacterial burden in the nose was higher for the 60B strain compared with the 60CSF strain (**Figure 5**; *P* < 0.0001). At 48 h post challenge, there were no observed significant differences between the two strains in any niche (data not shown).

**Figure 6:**
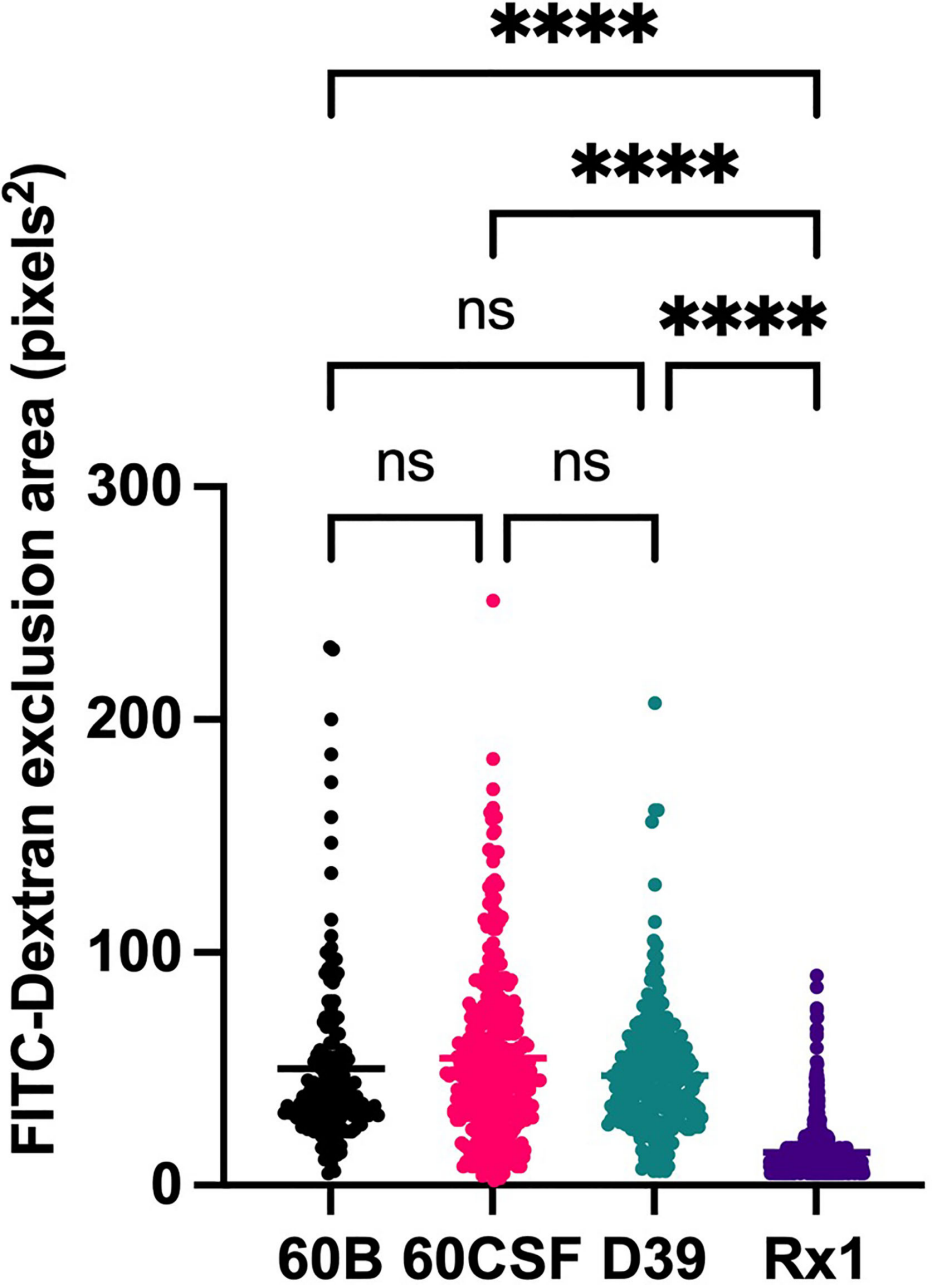
Capsule area of blood and CSF isolates, measured using FITC-dextran exclusion assay. *S. pneumoniae* D39, Rx1, serotype 15C ST8711 blood isolate (60B) and CSF isolate (60CSF) were grown overnight before being resuspended to an OD_600_ of 0.6 in 1 mL PBS. 80µL of bacterial suspension was added to 20µL FITC-dextran (10mg/mL in MilliQ water). A volume of 10 µL of the bacterial FITC-dextran suspension was pipetted onto microscope slides, fixed with a coverslip, and imaged using a laser scanning microscope. Statistical analysis was performed using Kruskal-Wallis test; ****P < 0.0001. Imaging was conducted on 3 separate days with fresh bacterial suspensions prepared each time. ns, not significant.

**Figure 7:**
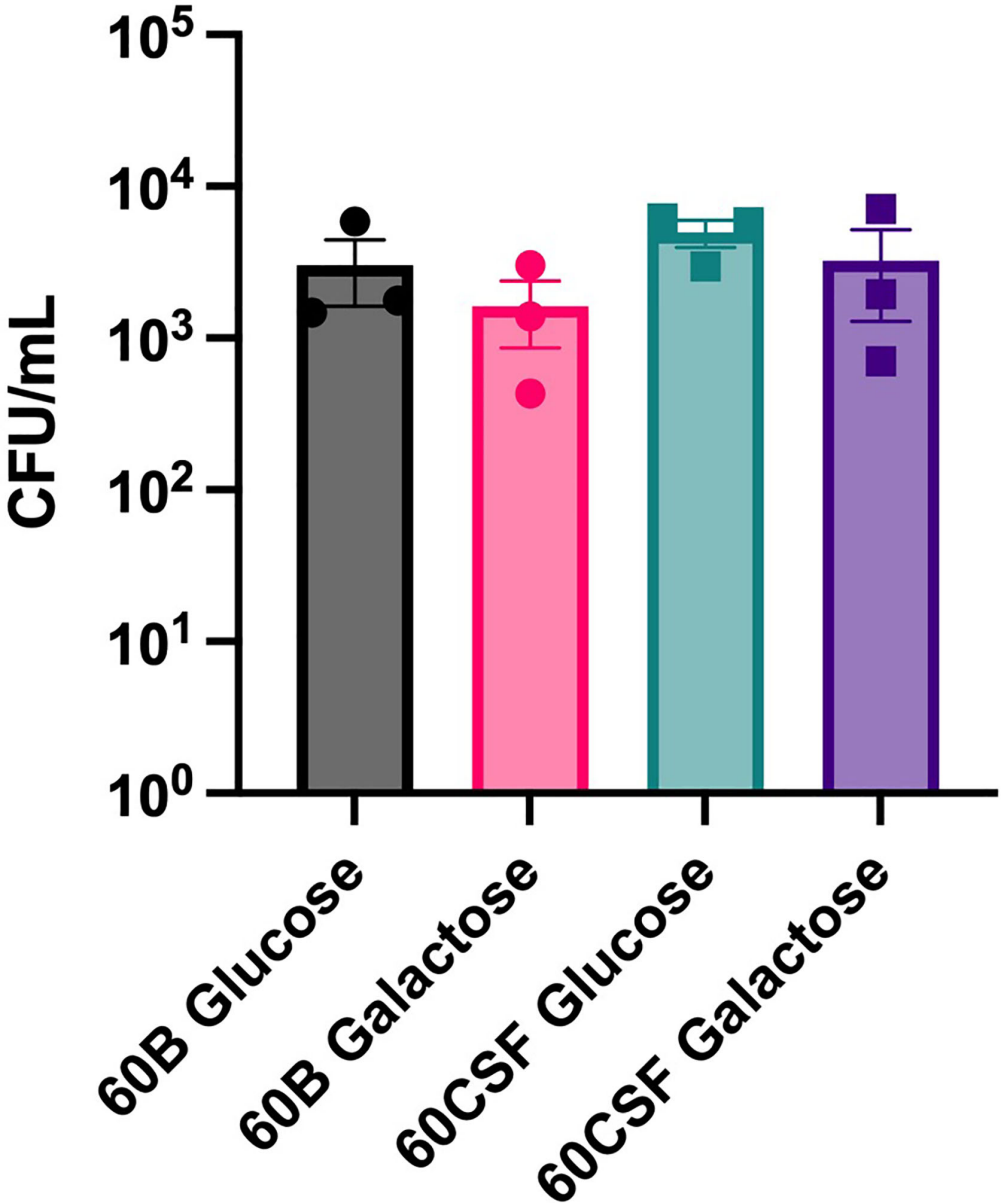
Adherence of 60B and 60CSF to Detroit 562 cells. *S. pneumoniae* strains 60B and 60CSF at OD_600_ 0.2 were inoculated onto monolayers of Detroit 562 cells in CDM + glucose or CDM + galactose, and incubated for 2 h before being tested for adherent bacteria. Data are mean adherent bacteria ± standard error mean (SEM) from three independent assays, each performed in triplicate. Statistical analysis was performed using two-tailed Student’s *t* test. No significant differences were detected between the adherence of the strains in different conditions.

**Figure 8:**
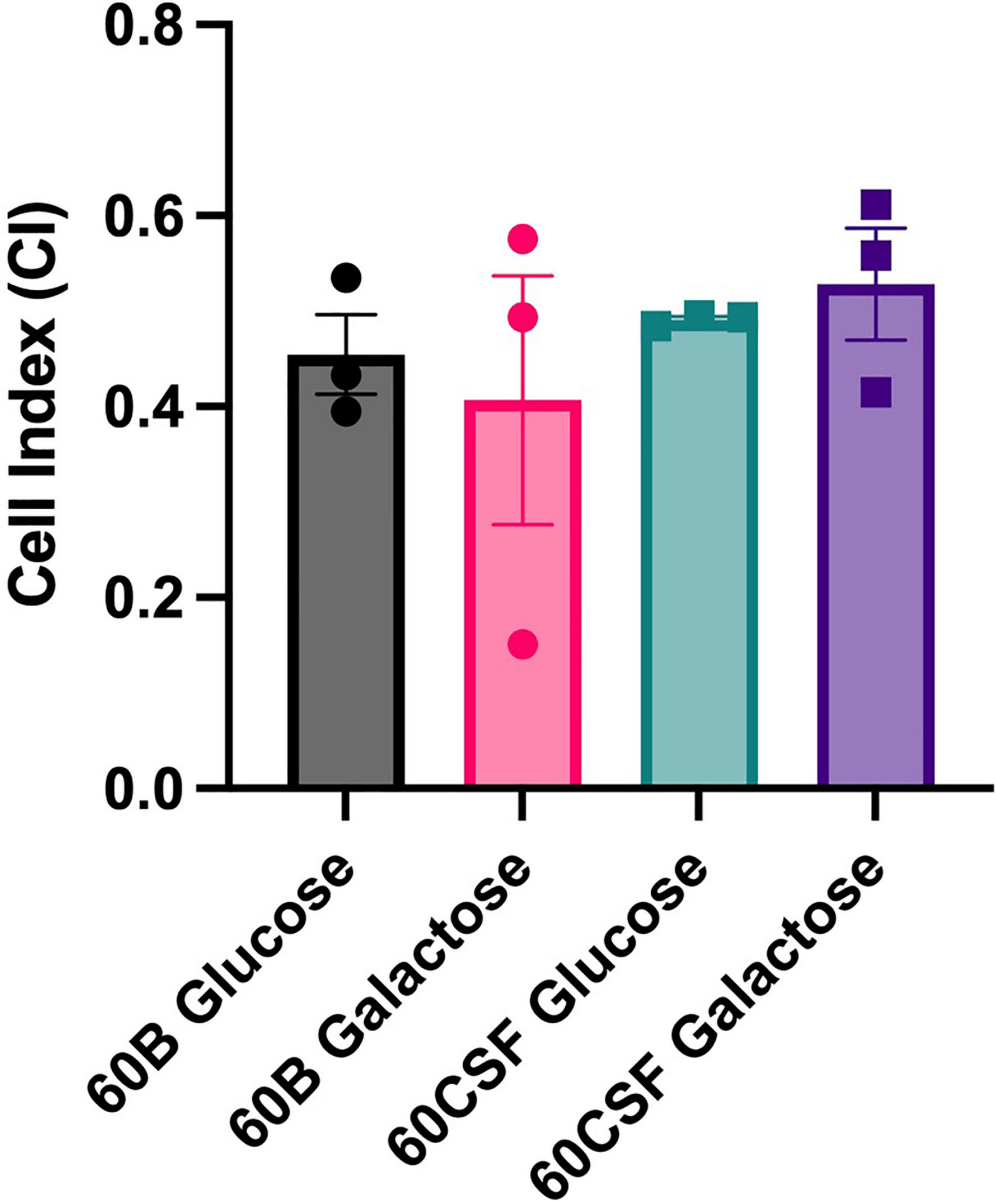
Maximum biofilm formation by 60B and 60CSF. 60B and 60CSF were cultured overnight on BA plates before being diluted to a final OD_600_ of 0.05 in CDM + 0.5% glucose or CDM + 0.5% galactose. 200 µL of each bacterial strain in each sugar was placed into wells of a xCELLigence E-plate, with the plate being placed in the cradles of the RTCA-DP platform, and incubated at 37°C. Biofilm formation was determined by measuring cell index (CI) every 15 min over 24 h using real-time cell analysis (RTCA) xCELLigence technology. Data are mean maximum CI ± standard error mean (SEM) from three independent assays, each performed in triplicate. Statistical analysis was performed using two-tailed Student’s *t* test. No significant differences were detected between the biofilm formation of the strains in different carbon sources.

Given the observed niche preferences for the strains at the 24 h time point, where the 60B strain was higher in the nose, while 60CSF higher in the ear, we investigated whether these strains produced varied amounts of capsule that may account for these differences. We analyzed the relative capsule abundance by measuring the FITC-Dextran exclusion area of the two isolates, as well as D39 and Rx1 as encapsulated and unencapsulated control strains, respectively. No significant difference in the mean FITC-Dextran exclusion area was observed between the 60B and 60CSF strains (**Figure 6**). The unencapsulated control strain Rx1 displayed a significantly lower mean capsule area compared with the D39 strain (*P* < 0.01), which showed a mean area comparable to that of the 60B strain, while there was a minor but significant difference compared with the 60CSF strain (**Figure 6**; *P* < 0.01). We next considered whether the difference in tissue tropism could be due to an altered capacity of the bacterial strains to adhere to the nasopharyngeal epithelium. The *in vitro* adherence of each strain to Detroit 562 pharyngeal cells was explored in the presence of glucose or galactose as the sole carbon source, the latter representing the prevalent sugar present in the nasopharynx (Paixão et al., 2015). No significant difference in the total number of adherent cells was observed between the strains in either carbon source (**Figure 7**). Together, these data suggest that the differences in tissue tropism are not merely a consequence of altered capsule expression or nasopharyngeal adherence capabilities between the strains. To determine whether the 60B and 60CSF strains displayed a difference in biofilm formation capacity, we performed an assessment of their biofilm formation with the xCELLigence instrument. An analysis of biofilm formation was performed in glucose, the preferred carbon source, and galactose, a carbon source accessible during nasopharyngeal colonization, where biofilm formation is thought to occur. No significant difference in the rate of biofilm formation over a 24h period was observed between the strains (data not shown). Furthermore, although the maximum biofilm formation in 60CSF appeared higher than in 60B, with higher biofilm levels in the galactose medium, this difference was not statistically significant (**Figure 8**).

### Genomic Analyses Identify a Putative Alpha-Galactosidase That May Contribute to Phenotypic Differences Between the Blood and CSF Strains

**Figure 9:**
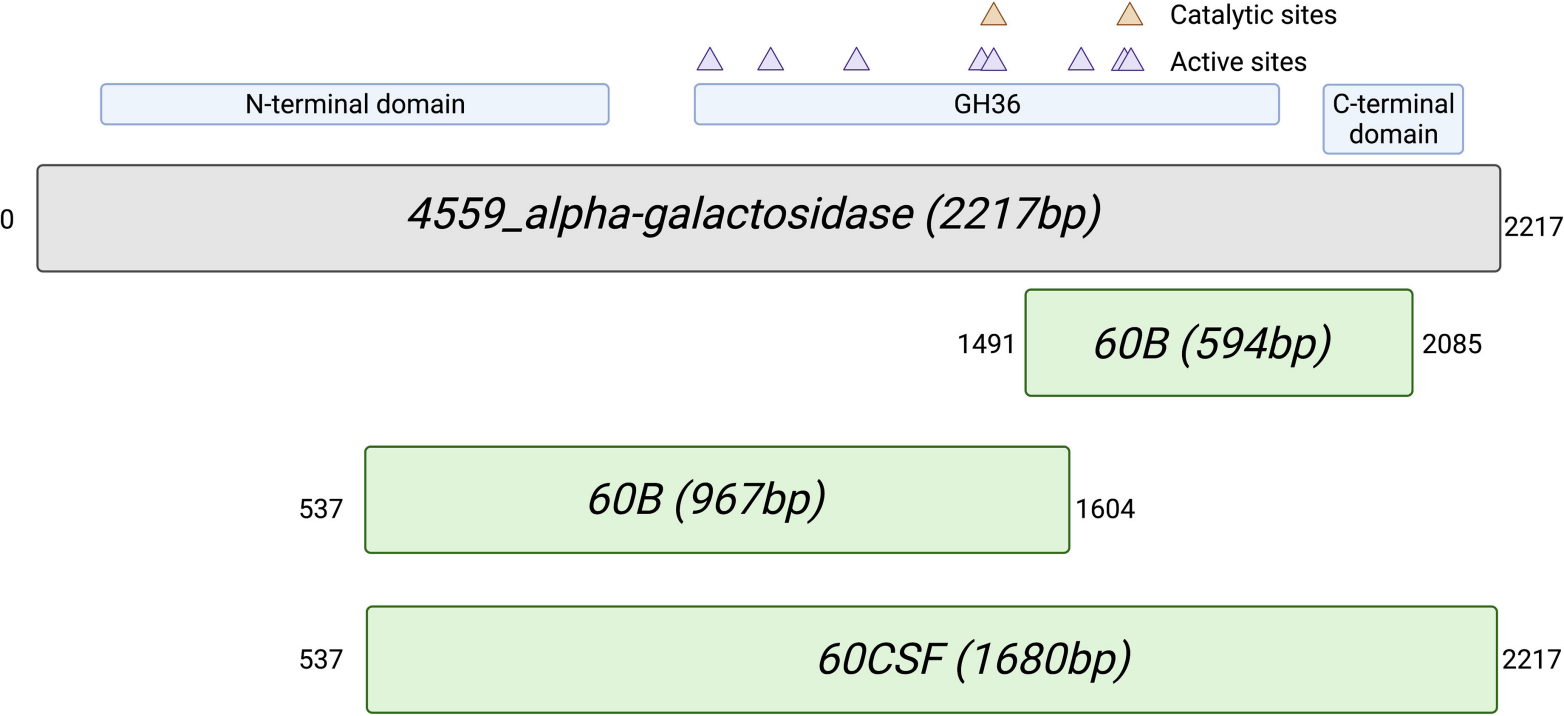
Alignment of the alternative alpha-galactosidase gene with putative gene fragments. Gene fragments of 60B and 60CSF that correspond to an alternative alpha-galactosidase (protein ID: WP_000158269.1; locus tag: FJH52_RS10440; gene ID: GI:446080414) found in *S. pneumoniae* strain 4559. The blood isolate, 60B, contained fragments that were 594bp and 967bp in length, whilst the CSF isolate, 60CSF, contained a single fragment of 1680bp. Numbers outside of gene fragments correspond to nucleotide position within strain 4559 alpha-galactosidase. A conserved domain search was performed on using NCBI conserved domain search (Lu et al., 2020). A glycosyl hydrolase family 36 (GH36) motif was found in strain 4559 alternative alpha-galactosidase. Two aspartic acid residues have been identified as the catalytic nucleophile and the acid/base, respectively, as indicated by the orange triangles. Active sites within the GH36 motif are indicated by purple triangles. A glycosyl hydrolase family 36 N-terminal domain, with a beta-supersandwich fold, and a glycosyl hydrolase family 36 C-terminal domain, with a beta-sandwich structure with a Greek key motif, were also located within the strain 4559 alternative alpha-galactosidase.

**Figure 10:**
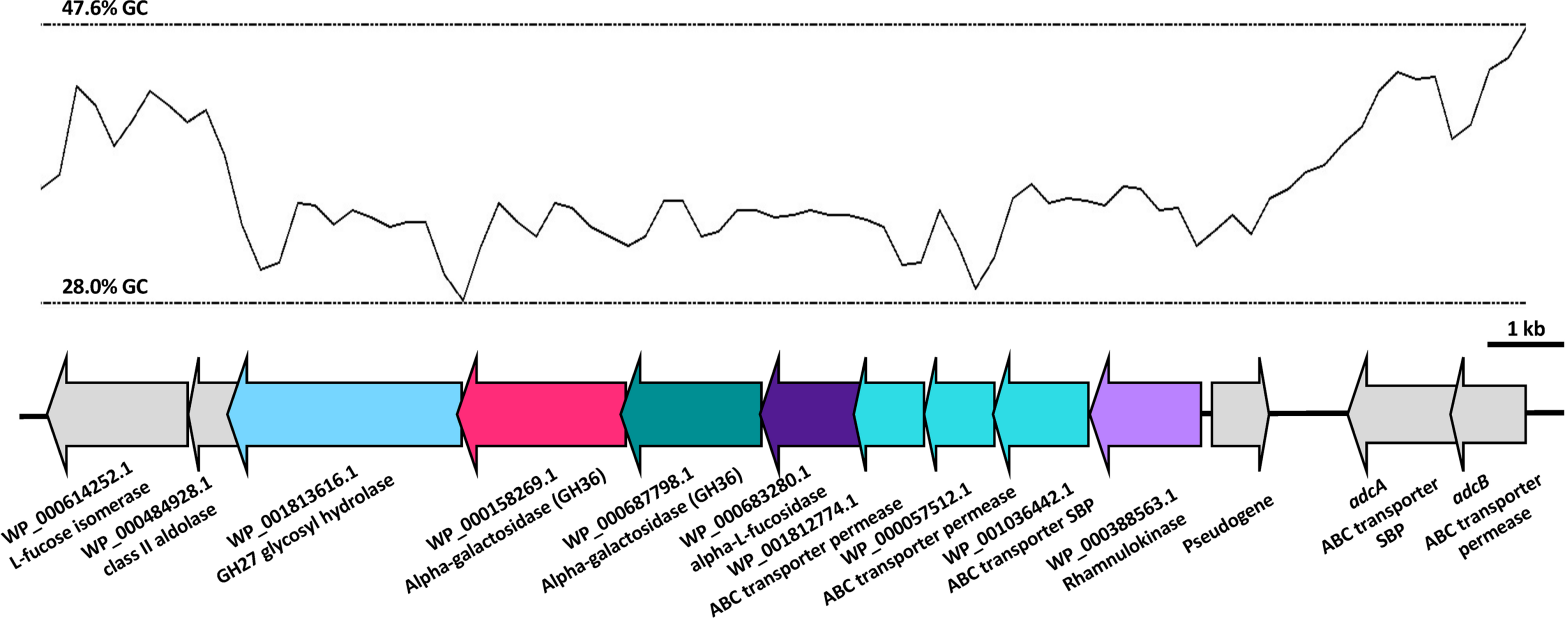
Organisation of the *S. pneumoniae* 4559 alpha-galactosidase region. The GC content of the DNA is shown as a black line, with the upper and lower limits for this region as dotted lines. Open reading frames of genes are depicted in the direction of transcription, with genes in the lower GC region shown as colored arrows and genes adjacent to this region as grey arrows. The protein identification (ID) or name (if known), and putative function are indicated below the genes. Genes have been drawn to scale.

As we have previously shown niche-adaptation differences between closely related isolates (Minhas et al., 2019) and isolates from the same patient (Tikhomirova et al., 2021) to be driven by a SNPs in loci responsible for uptake and utilization of raffinose, we performed whole genome sequencing of 60B and 60CSF on the Illumina platform, to determine whether the phenotypic differences observed here were a product of similar genomic events. SNP analysis comparing 60B and 60CSF sequences revealed that no SNPs were present in the well characterized raffinose locus or in any other coding regions. However, reciprocal best hit-based analysis revealed that there may be different fragments of a gene, encoding a putative glycosyl hydrolase family 36 (GH36) alpha-galactosidase, present between the blood and CSF isolates. The nucleotide and amino acid sequences shared no sequence similarity with the *aga* gene sequenced at the *raf* locus of these strains. We performed a BLASTn search using the nucleotide sequence for the largest fragment from the CSF isolate. This region showed 100% sequence identity to the latter 75% of an alpha-galactosidase gene that was present as part of the accessory genome in 68 out of 9029 other pneumococcal strains (0.75%), including the *S. pneumoniae* 4559, EF3030, and 947 strains (Junges et al., 2019; Minhas et al., 2019). Based on the short-read sequencing, obtained with the Illumina platform, the blood isolate appeared to possess two fragments spanning a 594 bp and 967 bp region of the alpha-galactosidase (**Supplementary Figure 2**), while the CSF isolate possessed one larger gene fragment encompassing 1680 bp in total (**Figure 9**). The sequencing did not identify regions with similarity to the first 537 bp of this full-length gene in either isolate. However, the fragments in the CSF isolate encompassed the remainder of the gene, while the contigs from the Illumina sequencing did not yield sequences that correspond to the final 132 bp of this gene for the 60B strain. We conducted further analyses of the alpha-galactosidase gene in the *S. pneumoniae* 4559 blood isolate. The alpha-galactosidase gene, depicted as a pink arrow (Protein ID WP_000158269.1; **Figure 10**), was present in a ~13 kb region of DNA that possessed a relatively low GC content (33% GC) to that of the upstream and downstream DNA (40.4% GC and 40% GC, respectively), suggesting a possible horizontal gene transfer event may have occurred. However, there were no other genes or features identified nearby that would indicate that this region was a mobile genetic element. This ~13 kb region of *S. pneumoniae* 4559 contained nine genes in total, including genes with putative functions such as a NPCBM/NEW2 domain-containing glycoside hydrolase family 27 protein (WP_001813616.1), an additional alpha-galactosidase (WP_000687798.1), an alpha-L-fucosidase (WP_000683280.1), two carbohydrate ABC transporter permeases, (WP_001812774.1 and WP_000057512.1), an ABC transporter substrate binding protein (WP_001036442.1), and a rhamnulokinase (WP_000388563.1) (**Figure 10**). In *S. pneumoniae* 4559, this region was situated adjacent to the zinc acquisition pathway genes, *adcCBA*. We then examined whether either the 60B or 60CSF contigs contained the adjacent gene sequences. Both isolates contained contigs with sequence identity to the glycoside hydrolase family 27 and alpha-galactosidase genes.

## Discussion

Over the past 20 years marked reductions in total death rates were achieved for many of the world’s most important communicable diseases including malaria, tuberculosis and diarrhea, but the mortality due to lower respiratory infections and meningitis remained fairly constant (GBD 2015 Child Mortality Collaborators, 2016). Notably, the decrease in pneumococcal isolates with serotypes covered by currently available conjugate vaccines, which often cause bacteremic disease, was counterbalanced by an increase in cases of meningitis associated with strains less likely to cause bacteremia (Mukerji and Briles, 2020). The lack of reduction in death rates of the diseases caused by the pneumococcus, despite the availability of an effective vaccine and suitable antimicrobial therapy, indicates that invasive pneumococcal disease is less well understood than we thought.

Understanding the bacterial features that dictate disease progression and phenotype is complicated by the high genetic diversity of *S. pneumoniae*. Certain serotypes have been associated with specific disease types, but non-capsular differences also contribute to disease progression. We have previously identified genetic and metabolic differences between clinical isolates of the same sequence type that were derived from the blood and ear of different patients. While these isolates exhibited related genetic backbones, these strains were isolated from distinct patients and possessed many additional gene alterations. In this study, we explored two clinical strains, possessing identical serotype, sequence type, and antibiotic sensitivity profiles, isolated at the same time from the blood and CSF of a single pediatric patient presenting with meningitis. These strains provided a unique opportunity to investigate whether differences may have arisen during the course of disease that could contribute to a change in lifestyle, such as permitting transfer across the blood-brain barrier or altered survival in the brain and CSF environment.

*In vitro* phenotypic characterization of these strains identified major differences in the metabolic capacity of the blood and CSF isolates. Compared with the isolate from the CSF, the blood isolate was unable to metabolize a range of carbohydrates, including L-rhamnose, 2-deoxy-D-ribose, D-arabinose, chondroitin sulfate c, D-tagatose, turanose and dihydroxy acetone and the two alpha-galactoside sugars, raffinose and melibiose. Importantly, we found that the blood isolate was unable to grow when raffinose was the sole carbon source.

This is distinct from our previous findings for serotype 14 ST15 strains, in which a pneumococcal blood isolate was able to grow in raffinose, while an ear isolate displayed perturbed growth (Minhas et al., 2019). In the current study, the CSF and blood isolates appeared to possess extensive genomic similarity, with short-read genomic analyses identifying no SNPs in the coding regions of genes between these two strains. However, we found that despite the observed capacity for raffinose to be metabolized and sustain growth of the CSF isolate, both of the strains possessed a premature stop codon in the *aga* gene. The impact of the premature stop codon on the stability of the *aga* mRNA is unclear, but a truncated protein lacking the C-terminal region that harbors the catalytic and active sites suggest this protein would lack functionality. While the nucleotide sequence of this *aga* gene at the *raf* locus most closely resembled the D39 (SPD_1678) and TIGR4 (SP_1898) sequences, we also identified fragments of an alternative alpha-galactosidase gene region that was differentially present in the two strains. These fragments shared 100% identity with a portion of a gene encoding an alternative alpha-galactosidase that was present in strains including *S. pneumoniae* strain 4559, EF3030, 947, and 70585.

We performed a conserved domain search to determine whether the C-terminal region of this protein, which was not detected in the blood isolate, might be of functional significance. The region missing from the larger fragment of the blood isolate corresponded to a putative glycosyl hydrolase family 36 (GH36) motif, which contains three out of the eight putative active site residues, including one of the catalytic aspartic acid residues. By comparison, the first 538 bp region (~179 N-terminal amino acids) that were not detected in either isolate did not contain any of the putative active site residues. The gene regions identified did not possess significant similarity to any other related Streptococcal species, but the identification of similarity in other pneumococcal isolates points to the acquisition of this gene through a recombination event with another pneumococcal strain, possibly during nasopharyngeal colonization. In *S. pneumoniae* 4559, the sequence of DNA including the adjacent genes possesses a lower GC content, which suggests that it may have originally been acquired from another species with a relatively lower GC%. Despite the absence of features consistent with a mobile genetic element, the pneumococcus is naturally transformable and will readily incorporate DNA from the environment. However, both the 60B and 60CSF appear to possess sequences that align with the genes upstream and downstream of this alpha-galactosidase. As a result, these findings must be validated by long-read sequencing to have confidence in the sequence and precise location of this region in both strains. Nevertheless, as these isolates were obtained from different sites of the same patient, it is possible that after transition to the brain, the 60B strain lost the C-terminal region of this additional alpha-galactosidase gene, resulting in abolished raffinose utilization capacity.

Notwithstanding the above differences in the *aga* gene sequences, the blood isolate also showed reduced transcription of *aga* at the *raf* locus, as well as *rafK*, which encodes the ATP-binding component of the raffinose importer. No difference in expression was observed for the permease component (*rafG*), but as RafK is required to energize the transport of raffinose by this pathway, these data point to a likely reduction in the import of raffinose in the isolate from the blood compared with the CSF isolate, which may further compound growth defect and reduced transcription of the *rafK* gene observed. Unexpectedly, we found that the expression of the gene encoding the transcriptional activator of these operons, *rafR*, was undetectable in both isolates. The *rafR* gene is co-transcribed with *rafS*, and their gene products regulate the raffinose-dependent stimulation of a divergently transcribed second promoter (*P_A_)* directing the expression of *aga* (Rosenow et al., 1999). As a result, the regulation of this operon may be largely dependent on the levels of raffinose in the cell. Thus, the observed reduced transcription of the raffinose uptake genes in the blood isolate may be a consequence of increased accumulation of raffinose due to the potential inactivity of the alpha-galactosidase, whereby excess uncleaved raffinose may directly interact with the regulatory protein, leading to repression of these genes in the blood isolate, compared with the CSF isolate.

In addition to the metabolic differences observed *in vitro*, these strains possessed markedly different virulence profiles in mice. No mice challenged with the blood isolate had detectable levels of pneumococci in the ears, while we recovered the CSF isolate from the ears of >85% of the infected mice. In this study, the blood isolate displayed an inability to transit to the ear after intranasal challenge. These data are consistent with previous findings in other serotypes and sequence types, in which bacteria isolated from the blood typically display lower bacterial loads in the ears of mice post-challenge, compared with bacteria from other isolation sites (Amin et al., 2015; Minhas et al., 2019). In our previous study, increased capacity to metabolize raffinose correlated with reduced ear titers, but the defect in raffinose utilization was attributed to SNPs present in the regulator RafR or the import protein component RafK (Minhas et al., 2019). Another distinction between this study and the previous is that the isolates were obtained from the same patient. Although it is not known whether the blood strain originated from the CSF isolate or vice versa, this approach resulted in substantially reduced background genetic differences, including an absence of detectable SNPs. In this study, the inability of the blood strain to utilize raffinose is likely a consequence of impaired alpha-galactosidase activity and is correlated with reduced ear titers. Together, these data suggest that perturbation of this pathway at distinct stages may result in substantially different virulence phenotypes. The burden of bacteria in the nasopharynx was higher for the blood isolate than the CSF isolate, suggesting that a defect in alpha-galactosidase activity may favour survival in this niche, or perturb transit to alternate sites. Overall, these findings expand our understanding of the pneumococcal factors that may dictate disease types in humans, pointing to possible new features of clinical significance.

The basis for the involvement of a raffinose utilization pathway in pneumococcal virulence remains unclear, but there are two prevailing hypotheses. First, that pneumococci may be exposed to raffinose in the oropharynx from dietary sources (generally plants). Consistent with this, many other streptococcal species, including *Streptococcus mutans*, and lactic acid bacteria possess loci with similar genes and gene organization to the pneumococcal raffinose locus. Alternatively, this pathway may function to import and utilize structurally related human glycans. Aga has been shown to effectively cleave a galactose unit from a linear blood group antigen, suggesting a possible alternative physiological substrate of this pathway, but as recent studies showed that Aga is localized intracellularly, the role of this protein in host glycan processing is also dependent on the efficient uptake of the glycan from the environment (Hobbs et al., 2019). However, these studies were performed on the Aga protein from the TIGR4 strain, which possesses limited (44%) amino acid sequence identity to the alternative alpha-galactosidase discussed here. While humans lack the capacity to metabolize raffinose, there is evidence that pneumococci require this pathway during disease, as detectable expression of the *raf* genes was observed in bacteria isolated from the lungs of mice (Minhas et al., 2020). Further studies of the full-length and truncated variants of this alternative alpha-galactosidase enzyme are required to elucidate the potential substrates and possible role *in vivo*. It is possible that the alpha-galactosidase variant described herein displays an altered specificity for an as yet unidentified substrate that is crucial during disease.

In a previous study, we found that during infection of the host, *S. pneumoniae* display two main patterns of gene expression; one was characteristic of bacteria in blood and one of bacteria in tissue, such as lungs and brain (Oggioni et al., 2006). In the present study, two clinical strains originated from the blood (planktonic state) and the brain (biofilm state) of a 2-year-old child admitted to hospital with suspected meningitis, showed the same capability to form *in vitro* biofilm. Biofilm formation is one of the most important virulence traits for the bacteria. Biofilms act as a reservoir for organisms, enabling escape from the action of host antibodies or antibiotics, as well as facilitating production of toxins and generation of resistant organisms. Bacteria in biofilms may also detach and cause bloodstream infections. The biofilm state is often associated with solid-tissue related infections; in cystic fibrosis, chronic lung infections are due to *P. aeruginosa*, *Staphylococcus aureus* and *Haemophilus influenzae*, which reside in the airway in biofilms (Smyth, 2006; Starner et al., 2006). Otitis media is caused by nontypeable *H. influenzae* and pneumococcus living within a biofilm (Murphy, 2003; Weimer et al., 2010; Cevizci et al., 2015) and finally, endocarditis is considered to be a biofilm-related infection caused by staphylococci or streptococci (Donlan and Costerton, 2002). However, recent clinical studies of purpura fulminans, a disease state associated with meningococcal sepsis, showed that bacteria are mostly grouped in microcolonies in these lesions (Harrison et al., 2002). Thus, a new aspect of biofilm formation that remains poorly characterized may explain the unexpected findings for biofilm capacity obtained with the blood isolate. Importantly, the CSF and blood strains used in this study, were collected from the same patient who was admitted to the hospital with suspected meningitis and it is unknown whether the blood stain originated from the CSF isolate or vice versa. While the hematogenous route was for many years believed to be the only mode for the bacteria to enter the CSF, clinical studies in adult and neonatal human meningitis have shown that *S. pneumoniae* can travel directly from the nasopharyngeal tissue to the brain without any bloodstream invasion (Garges et al., 2006). Experimental infections have confirmed that initial nasopharyngeal colonization could be followed by isolation of high number of bacteria from olfactory epithelium, brain, olfactory bulbs and trigeminal ganglia in the absence of bacteremia (Van Ginkel et al., 2003). *In vivo* imaging also showed that pneumococci were able to directly localize in mice to the olfactory bulb and the brain, in the absence of detectable bacteremia (Marra and Brigham, 2001).

*S. pneumoniae* is an important commensal resident of the human nasopharynx, and like other commensals in this niche, colonisation is dependent on the capacity to adhere to the epithelium and exist within the nasal mucus. In our murine model of infection, we found that the blood isolate was better able to survive in the nasopharynx of the mice compared to the CSF strain. However, when the adherence of each strain was explored using *in vitro* cultured human nasopharyngeal epithelial cells, no significant difference in the total number of adherent cells was observed between the strains. In the nasopharynx, bacteria are predominantly embedded in the mucin glycans and pneumococci possess several surface components, such as surface-located pneumococcal adherence and virulence protein A (PavA), PavB and enolase (Eno), that enable persistance in this niche for weeks or months. Thus, inconsistencies between *in vitro* and *in vivo* results are likely due to the difference in available carbohydrates and the lack of expression of key pneumococcal surface adhesins in the *in vitro* assays. These adhesive interactions with the epithelial surface may be needed not only for colonization, but also for the initial step in the invasion process.

The pathophysiology of pneumococcal meningitis is not fully understood. In this study, we found that strains isolated from the brain and the blood of a single paediatric patient with meningitis and bacteraemia, showed remarkable differences *in vitro* and *in vivo* assays. Importantly, we found that the CSF strain could metabolize raffinose, while the blood isolate lacks this ability. Progression from carriage to invasive disease exposes the pneumococcus to markedly different micro-environments to which the organism must adapt, and this might explain the loss or the acquisition of one gene or another. The exact role of raffinose in disease progression, particularly in meningitis, is still unclear and further studies using comparative genomic and transcriptomic analyses of other *S. pneumoniae* serotypes and/or other meningitis-causing bacteria, are needed.

## Data Availability Statement

The datasets presented in this study can be found in online repositories. The name of the repository and accession number can be found below: NCBI; PRJNA803929.

## Ethics Statement

Animal experiments were approved by the University of Adelaide Animal Ethics Committee.

## Author Contributions

Conceptualization, HA, EB, AT, JP, and CT. Methodology, HA, EB, KM, AT, and CT. Investigation, HA, EB, KM, AT, ML, and CT. Formal analysis, HA, EB, AT, JP, and CT. Data curation, HA, EB, AT, JP, and CT. Writing—original draft preparation. HA, EB, AT, JP, and CT. All authors have read and agreed to the published version of the manuscript.

## Funding

This work was supported by the National Health and Medical Research Council (NHMRC) Investigator grant 1174876 to JP, and by the Australian Research Council (ARC) Discovery Project DP190102980 to CT and JP.

## Conflict of Interest

The authors declare that the research was conducted in the absence of any commercial or financial relationships that could be construed as a potential conflict of interest.

## Publisher’s Note

All claims expressed in this article are solely those of the authors and do not necessarily represent those of their affiliated organizations, or those of the publisher, the editors and the reviewers. Any product that may be evaluated in this article, or claim that may be made by its manufacturer, is not guaranteed or endorsed by the publisher.
