# Supplementary material for: Streptococcus pneumoniae Strains Isolated From a Single Pediatric Patient Display Distinct Phenotypes
Source: Front Cell Infect Microbiol. 2022 Mar 31;12:866259. doi: 10.3389/fcimb.2022.866259 (PMC9008571; doi:10.3389/fcimb.2022.866259)
Supplement: Supplementary file 1 [file DataSheet_1.docx]

Supplementary Material

**Supplementary Figure 1: Sequence alignment of the *aga* gene in 60B and 60CSF.** Sanger sequencing was used to generate the *aga* sequence for both 60B and 60CSF. At position 638, C has changed to an A (bold font), resulting in a premature stop codon in both 60B and 60CSF. Underlined bases indicate the start codon and stop codons, including both the premature stop codon and stop codon for the full-length product.

60B_aga ATGGGAGTTAGGATAGAGAATAATCTATTTTATGTTGAGAGTAAAAATCTAAGTTTGATT 60

60CSF_aga ATGGGAGTTAGGATAGAGAATAATCTATTTTATGTTGAGAGTAAAAATCTAAGTTTGATT 60

************************************************************

60B_aga ATTGAAAATCGAAATGGCTACTTACTTTTGAAACATTTAGGAAAGACTATTAAGAACTAT 120

60CSF_aga ATTGAAAATCGAAATGGCTACTTACTTTTGAAACATTTAGGAAAGACTATTAAGAACTAT 120

************************************************************

60B_aga AAAGGTTCCAATAGTGTTTATGAACGAGACCATGCCTTTTCAGGAAATCCAACGGCTACT 180

60CSF_aga AAAGGTTCCAATAGTGTTTATGAACGAGACCATGCCTTTTCAGGAAATCCAACGGCTACT 180

************************************************************

60B_aga AATCGAACCTTTAGTTTAGATACTCAGCGACAGATTTTTGGACAACATGGCTTAGGAGAT 240

60CSF_aga AATCGAACCTTTAGTTTAGATACTCAGCGACAGATTTTTGGACAACATGGCTTAGGAGAT 240

************************************************************

60B_aga TTTAGGAAACCAACCATACAGGTTCAGCATAGTGTAACTGAAGTAACAGACTTTCGATTT 300

60CSF_aga TTTAGGAAACCAACCATACAGGTTCAGCATAGTGTAACTGAAGTAACAGACTTTCGATTT 300

************************************************************

60B_aga GTAGAAGCAAAGATTTTAAAAGGTCAGAATGGTCCACAGGGCTTACCTTCTCCACATAGC 360

60CSF_aga GTAGAAGCAAAGATTTTAAAAGGTCAGAATGGTCCACAGGGCTTACCTTCTCCACATAGC 360

************************************************************

60B_aga ATGGACGATACAGAGACTCTTGTCTTAATGTTAGAAGATTCTAAGGCTCAACTTAGTCTG 420

60CSF_aga ATGGACGATACAGAGACTCTTGTCTTAATGTTAGAAGATTCTAAGGCTCAACTTAGTCTG 420

************************************************************

60B_aga ACTTTGTATTATACTACTTTTAATAATGATGCGACTATTGCTAGCTACAGTAAATTAGAT 480

60CSF_aga ACTTTGTATTATACTACTTTTAATAATGATGCGACTATTGCTAGCTACAGTAAATTAGAT 480

************************************************************

60B_aga AATAATAGTAATCAGGAAGTTGTCATCCATAAAGATTTTTCTTTTATGGCTGATTTTCCA 540

60CSF_aga AATAATAGTAATCAGGAAGTTGTCATCCATAAAGATTTTTCTTTTATGGCTGATTTTCCA 540

************************************************************

60B_aga GCTGCAGATTACGAAATAGTAACTCTGCAGGGTGCTTATGCTCGTGAAAAGACTGTTAGA 600

60CSF_aga GCTGCAGATTACGAAATAGTAACTCTGCAGGGTGCTTATGCTCGTGAAAAGACTGTTAGA 600

************************************************************

60B_aga CGTCAACAGGTAGAACAAGGAATCTTTTCGATTAGCT**A**AAACCGAGGTGCTTCTGGTCAT 660

60CSF_aga CGTCAACAGGTAGAACAAGGAATCTTTTCGATTAGCT**A**AAACCGAGGTGCTTCTGGTCAT 660

****************************************************************

60B_aga GCTCAAACACCAGCTCTTCTACTATGCGAACAAGGAGTCACAGAGGATGCTGGGAATGTG 720

60CSF_aga GCTCAAACACCAGCTCTTCTACTATGCGAACAAGGAGTCACAGAGGATGCTGGGAATGTG 720

************************************************************

60B_aga TTTGCTATTCAACTAATGTATAGTGGCAACTTTGAAGCTTTTGTTCAAAAAAATCAATTG 780

60CSF_aga TTTGCTATTCAACTAATGTATAGTGGCAACTTTGAAGCTTTTGTTCAAAAAAATCAATTG 780

************************************************************

60B_aga AATGAAGTTCGGGTGGCTATTGGCATTAATCCAGAAAACTTTTCTTGGAAGTTAGCTCCT 840

60CSF_aga AATGAAGTTCGGGTGGCTATTGGCATTAATCCAGAAAACTTTTCTTGGAAGTTAGCTCCT 840

************************************************************

60B_aga GAGGAATACTTTGAAACACCGGTAGCTTTAGTGACCCATTCAGATCAGGGATTAACTGGT 900

60CSF_aga GAGGAATACTTTGAAACACCGGTAGCTTTAGTGACCCATTCAGATCAGGGATTAACTGGT 900

************************************************************

60B_aga ATTAGTCATGAAAGTCAGAATTTTGTACTGAAGCACATTATGCTAAGTGAATTTTCTAAA 960

60CSF_aga ATTAGTCATGAAAGTCAGAATTTTGTACTGAAGCACATTATGCTAAGTGAATTTTCTAAA 960

************************************************************

60B_aga AAAGAACGTCCAATTCTAATCAATAACTGGGAAGCTACTTACTTTGACTTTCAGAGAGAA 1020

60CSF_aga AAAGAACGTCCAATTCTAATCAATAACTGGGAAGCTACTTACTTTGACTTTCAGAGAGAA 1020

************************************************************

60B_aga AAACTGTTAGAGTTAGCAGATGAAGCTAAGAAAGTTGGCATTGAACTTTTTGTATTAGAT 1080

60CSF_aga AAACTGTTAGAGTTAGCAGATGAAGCTAAGAAAGTTGGCATTGAACTTTTTGTATTAGAT 1080

************************************************************

60B_aga GATGGTTGGTTTGGCAATCGTTTTGATGATAATCGTGCTTTAGGTGATTGGGTTGTTAAT 1140

60CSF_aga GATGGTTGGTTTGGCAATCGTTTTGATGATAATCGTGCTTTAGGTGATTGGGTTGTTAAT 1140

************************************************************

60B_aga GAGGAAAAACTGGGTGGAAGTCTAGAAAGTCTGATTTCAGCTATCCATGAAAGAGGTTTG 1200

60CSF_aga GAGGAAAAACTGGGTGGAAGTCTAGAAAGTCTGATTTCAGCTATCCATGAAAGAGGTTTG 1200

************************************************************

60B_aga CAGTTTGGACTTTGGTTAGAACCCGAAATGATTTCTGTAGATAGTGATTTGTATCGTCAA 1260

60CSF_aga CAGTTTGGACTTTGGTTAGAACCCGAAATGATTTCTGTAGATAGTGATTTGTATCGTCAA 1260

************************************************************

60B_aga CATCCTGACTGGGCTATTCAGGTTCCTGGCTATGAGCATACTTATTCTCGGAATCAATTA 1320

60CSF_aga CATCCTGACTGGGCTATTCAGGTTCCTGGCTATGAGCATACTTATTCTCGGAATCAATTA 1320

************************************************************

60B_aga GTACTTAATCTTGCCAATCCTCAGGTAGTAGAATACTTGAAAAGTGTCTTAGATCAACTC 1380

60CSF_aga GTACTTAATCTTGCCAATCCTCAGGTAGTAGAATACTTGAAAAGTGTCTTAGATCAACTC 1380

************************************************************

60B_aga CTATCTTATCATGATATTGATTACATTAAATGGGATATGAACCGCAATATCACTAAGCTA 1440

60CSF_aga CTATCTTATCATGATATTGATTACATTAAATGGGATATGAACCGCAATATCACTAAGCTA 1440

************************************************************

60B_aga GGGAATGGATTAACTTATCTAGAGACACAGATGCAATCTCATCAGTACATGCTGGGGCTT 1500

60CSF_aga GGGAATGGATTAACTTATCTAGAGACACAGATGCAATCTCATCAGTACATGCTGGGGCTT 1500

************************************************************

60B_aga TACGAACTCGTTTCTTATCTGACAGAGAAGCACAGCCATATTCTCTTTGAGTCCTGCTCT 1560

60CSF_aga TACGAACTCGTTTCTTATCTGACAGAGAAGCACAGCCATATTCTCTTTGAGTCCTGCTCT 1560

************************************************************

60B_aga GGTGGTGGTGGACGAAATGATCTTGGTATGATGCGCTATTTCCCACAAGTCTGGGCTAGT 1620

60CSF_aga GGTGGTGGTGGACGAAATGATCTTGGTATGATGCGCTATTTCCCACAAGTCTGGGCTAGT 1620

************************************************************

60B_aga GATAATACAGATGCTATTGCACGTTTACCAATTCAATACGGTTCATCCTATCTCTATCCA 1680

60CSF_aga GATAATACAGATGCTATTGCACGTTTACCAATTCAATACGGTTCATCCTATCTCTATCCA 1680

************************************************************

60B_aga ACCATTTCTATGGGGGCTCATGTGTCAGCAGTACCGAATCATCAGATGGGGCGAACGACA 1740

60CSF_aga ACCATTTCTATGGGGGCTCATGTGTCAGCAGTACCGAATCATCAGATGGGGCGAACGACA 1740

************************************************************

60B_aga CCATTAGAAACACGTGGCCTTGTAGCAATGATGGGAAATTTGGGCTATGAACTTGATTTG 1800

60CSF_aga CCATTAGAAACACGTGGCCTTGTAGCAATGATGGGAAATTTGGGCTATGAACTTGATTTG 1800

************************************************************

60B_aga ACAAATTTATCAGATGAAGAGAAAGCTACGATTGCTAATCAGGTGAACTTGTATAAAGAA 1860

60CSF_aga ACAAATTTATCAGATGAAGAGAAAGCTACGATTGCTAATCAGGTGAACTTGTATAAAGAA 1860

************************************************************

60B_aga TTACGACCAGTAGTTCAGTTAGGACAACAGTATAGACTAATTAATCCTGATACTGCATCC 1920

60CSF_aga TTACGACCAGTAGTTCAGTTAGGACAACAGTATAGACTAATTAATCCTGATACTGCATCC 1920

************************************************************

60B_aga AATGAAGCTGCTGTACAATTTAATTACAAAAATCAAACGATTGTAACCTACGTTCGCGTT 1980

60CSF_aga AATGAAGCTGCTGTACAATTTAATTACAAAAATCAAACGATTGTAACCTACGTTCGCGTT 1980

************************************************************

60B_aga TTATCTGTTGTAGAGACCATGGAAACAACTTTAAAGTTAAAAGATTTGGATGAAGAGGGA 2040

60CSF_aga TTATCTGTTGTAGAGACCATGGAAACAACTTTAAAGTTAAAAGATTTGGATGAAGAGGGA 2040

************************************************************

60B_aga CTATATGAATTACAGGAAAATGGCGAAGTTTACTCAGGTGCAGAACTCATGTATGCGGGT 2100

60CSF_aga CTATATGAATTACAGGAAAATGGCGAAGTTTACTCAGGTGCAGAACTCATGTATGCGGGT 2100

************************************************************

60B_aga TTAACTGTTATTTTATCCCAAGGAGATTTTTTGAGTAAACAGTATATTTTTAGAAGACTA 2160

60CSF_aga TTAACTGTTATTTTATCCCAAGGAGATTTTTTGAGTAAACAGTATATTTTTAGAAGACTA 2160

************************************************************

60B_aga TAA 2163

60CSF_aga TAA 2163

***

**Supplementary Figure 2: Sequence alignment of the 60B and 60CSF *aga* gene fragments with *aga* in *S. pneumoniae* strain 4559.** Reciprocal best hit -based analysis on results from Illumina sequencing revealed *aga* gene fragments in 60B and 60CSF. These fragments did not correspond to the *aga* gene previously sequenced at the raf locus but instead shared 100% identity with a portion of a gene encoding an alternative alpha-galactosidase (2217bp) that was present in *S. pneumoniae* strain 4559 (labelled 4559_2217bp). The blood isolate, 60B, contained fragments that were 594bp and 967bp in length, whilst the CSF isolate, 60CSF, contained a single fragment of 1680bp.

60B_594bp ------------------------------------------------------------ 0

60B_967bp ------------------------------------------------------------ 0

60CSF_1680bp ------------------------------------------------------------ 0

4559_2217bp ATGACGATTTATATTAATAAGGACGAGACCGTTTTTCATTTGGCAATGAAAGATAGTAGT 60

60B_594bp ------------------------------------------------------------ 0

60B_967bp ------------------------------------------------------------ 0

60CSF_1680bp ------------------------------------------------------------ 0

4559_2217bp TATATTTTTAGAATTTTAGAAAATGGGGAACTTCAACATCTACATTTTGGGAAAAGGATT 120

60B_594bp ------------------------------------------------------------ 0

60B_967bp ------------------------------------------------------------ 0

60CSF_1680bp ------------------------------------------------------------ 0

4559_2217bp CATGTCAAGGAAAATTATAACCAATTGATGGCCTATGAAAAAAGAGGATTTGAAGTATCT 180

60B_594bp ------------------------------------------------------------ 0

60B_967bp ------------------------------------------------------------ 0

60CSF_1680bp ------------------------------------------------------------ 0

4559_2217bp TTTTCTGAAGAATTTGAGGATATTCAACAGTCTATGATACAAAATGAATATTCTTCATAT 240

60B_594bp ------------------------------------------------------------ 0

60B_967bp ------------------------------------------------------------ 0

60CSF_1680bp ------------------------------------------------------------ 0

4559_2217bp GGGAAAGGAGATTTTCGGCATCCAGCCTTTCAAGTTCAAGGAATGAATGGTAGTAGGATA 300

60B_594bp ------------------------------------------------------------ 0

60B_967bp ------------------------------------------------------------ 0

60CSF_1680bp ------------------------------------------------------------ 0

4559_2217bp ACGACACTAAAATATCAAGGTTTTGAACTTGAAAAAGGGAAAAATCGTCTTAACTCTCTA 360

60B_594bp ------------------------------------------------------------ 0

60B_967bp ------------------------------------------------------------ 0

60CSF_1680bp ------------------------------------------------------------ 0

4559_2217bp CCTTCAACATTTGATGATATTGGTCAGTGTGCGGAAACATTAACGATTATTTTAACAGAT 420

60B_594bp ------------------------------------------------------------ 0

60B_967bp ------------------------------------------------------------ 0

60CSF_1680bp ------------------------------------------------------------ 0

4559_2217bp TCCATATTAGATTTAACTGTTAGACTAAATTACACAATTTTTCCGGAATACAATGTCTTA 480

60B_594bp ------------------------------------------------------------ 0

60B_967bp ---------------------------------------------------------ATG 3

60CSF_1680bp ---------------------------------------------------------ATG 3

4559_2217bp GTTAGAAATACGGAATTTTTAAATAATAGCAATAATAAGTTGACTCTTTTGAAAGCAATG 540

60B_594bp ------------------------------------------------------------ 0

60B_967bp AGCTTACAGCTAGATCTACCTGATAGTCAATATGACTTTATTCAATTTTCTGGAGCATGG 63

60CSF_1680bp AGCTTACAGCTAGATCTACCTGATAGTCAATATGACTTTATTCAATTTTCTGGAGCATGG 63

4559_2217bp AGCTTACAGCTAGATCTACCTGATAGTCAATATGACTTTATTCAATTTTCTGGAGCATGG 600

60B_594bp ------------------------------------------------------------ 0

60B_967bp CTGAGGGAACGTCAGTTATATAGAACTTCGCTTAGACCAGGTATTCAAGCAATAGATAGC 123

60CSF_1680bp CTGAGGGAACGTCAGTTATATAGAACTTCGCTTAGACCAGGTATTCAAGCAATAGATAGC 123

4559_2217bp CTGAGGGAACGTCAGTTATATAGAACTTCGCTTAGACCAGGTATTCAAGCAATAGATAGC 660

60B_594bp ------------------------------------------------------------ 0

60B_967bp TTGAGATACTCATCAAGTCCTCAGCAAAATCCTTTCTTTATGCTATCAAGGAGGGAAACT 183

60CSF_1680bp TTGAGATACTCATCAAGTCCTCAGCAAAATCCTTTCTTTATGCTATCAAGGAGGGAAACT 183

4559_2217bp TTGAGATACTCATCAAGTCCTCAGCAAAATCCTTTCTTTATGCTATCAAGGAGGGAAACT 720

60B_594bp ------------------------------------------------------------ 0

60B_967bp ACAGAGCATAGTGGTGAGGTTTATGGTTTTAACTTTATCTATTCTGGAAATTTTCAAAAT 243

60CSF_1680bp ACAGAGCATAGTGGTGAGGTTTATGGTTTTAACTTTATCTATTCTGGAAATTTTCAAAAT 243

4559_2217bp ACAGAGCATAGTGGTGAGGTTTATGGTTTTAACTTTATCTATTCTGGAAATTTTCAAAAT 780

60B_594bp ------------------------------------------------------------ 0

60B_967bp ATGATTGAAGTTGACCATTTTGACACCGCTAGAGTAACGGTAGGAATAAATCCAGTAGAA 303

60CSF_1680bp ATGATTGAAGTTGACCATTTTGACACCGCTAGAGTAACGGTAGGAATAAATCCAGTAGAA 303

4559_2217bp ATGATTGAAGTTGACCATTTTGACACCGCTAGAGTAACGGTAGGAATAAATCCAGTAGAA 840

60B_594bp ------------------------------------------------------------ 0

60B_967bp TTTCGTTTTTTATTAAATCCTGCCGAAAGTTTTGTGACACCAGAAGCAATTGTGATCTAT 363

60CSF_1680bp TTTCGTTTTTTATTAAATCCTGCCGAAAGTTTTGTGACACCAGAAGCAATTGTGATCTAT 363

4559_2217bp TTTCGTTTTTTATTAAATCCTGCCGAAAGTTTTGTGACACCAGAAGCAATTGTGATCTAT 900

60B_594bp ------------------------------------------------------------ 0

60B_967bp TCTGATCAAGGGATGAATCAGATGAGCCAACAACTATCAGATTTTTATCGACATCATTTA 423

60CSF_1680bp TCTGATCAAGGGATGAATCAGATGAGCCAACAACTATCAGATTTTTATCGACATCATTTA 423

4559_2217bp TCTGATCAAGGGATGAATCAGATGAGCCAACAACTATCAGATTTTTATCGACATCATTTA 960

60B_594bp ------------------------------------------------------------ 0

60B_967bp GTTAATCCTAATTTTTCTCAAGCTAGTCGTCCTATAATACTCAATAGTTGGGAAACATTT 483

60CSF_1680bp GTTAATCCTAATTTTTCTCAAGCTAGTCGTCCTATAATACTCAATAGTTGGGAAACATTT 483

4559_2217bp GTTAATCCTAATTTTTCTCAAGCTAGTCGTCCTATAATACTCAATAGTTGGGAAACATTT 1020

60B_594bp ------------------------------------------------------------ 0

60B_967bp TATTTTGACTTGAGTACAGAAAAAATTTTAGATTTAGCAAAGGCTGCTAAAGATTTAGGG 543

60CSF_1680bp TATTTTGACTTGAGTACAGAAAAAATTTTAGATTTAGCAAAGGCTGCTAAAGATTTAGGG 543

4559_2217bp TATTTTGACTTGAGTACAGAAAAAATTTTAGATTTAGCAAAGGCTGCTAAAGATTTAGGG 1080

60B_594bp ------------------------------------------------------------ 0

60B_967bp ATAGAATTATTTGTACTGGATGATGGTTGGTTTGGTCATAGGAAAGATGACAAAAGTTCT 603

60CSF_1680bp ATAGAATTATTTGTACTGGATGATGGTTGGTTTGGTCATAGGAAAGATGACAAAAGTTCT 603

4559_2217bp ATAGAATTATTTGTACTGGATGATGGTTGGTTTGGTCATAGGAAAGATGACAAAAGTTCT 1140

60B_594bp ------------------------------------------------------------ 0

60B_967bp CTGGGTGATTGGGTAACAGATAGAAGTCGCCTTCCTGAAGGTATTGGATTTCTTGCAGAT 663

60CSF_1680bp CTGGGTGATTGGGTAACAGATAGAAGTCGCCTTCCTGAAGGTATTGGATTTCTTGCAGAT 663

4559_2217bp CTGGGTGATTGGGTAACAGATAGAAGTCGCCTTCCTGAAGGTATTGGATTTCTTGCAGAT 1200

60B_594bp ------------------------------------------------------------ 0

60B_967bp GAAATTCACAAAATAGGTTTACAATTTGGTTTGTGGTTTGAGCCTGAAATGATTTCTATT 723

60CSF_1680bp GAAATTCACAAAATAGGTTTACAATTTGGTTTGTGGTTTGAGCCTGAAATGATTTCTATT 723

4559_2217bp GAAATTCACAAAATAGGTTTACAATTTGGTTTGTGGTTTGAGCCTGAAATGATTTCTATT 1260

60B_594bp ------------------------------------------------------------ 0

60B_967bp GATAGTGATTTGTACAAGAATCATGCCGATTGGACTATCCATTTGTTAGACAGAGAGAAG 783

60CSF_1680bp GATAGTGATTTGTACAAGAATCATGCCGATTGGACTATCCATTTGTTAGACAGAGAGAAG 783

4559_2217bp GATAGTGATTTGTACAAGAATCATGCCGATTGGACTATCCATTTGTTAGACAGAGAGAAG 1320

60B_594bp ------------------------------------------------------------ 0

60B_967bp TCAGTAGGAAGAAATCAATATGTGTTGGATTTGACGAGACAGGAAGTTGTTGATTATCTT 843

60CSF_1680bp TCAGTAGGAAGAAATCAATATGTGTTGGATTTGACGAGACAGGAAGTTGTTGATTATCTT 843

4559_2217bp TCAGTAGGAAGAAATCAATATGTGTTGGATTTGACGAGACAGGAAGTTGTTGATTATCTT 1380

60B_594bp ------------------------------------------------------------ 0

60B_967bp TTTGATTCTATTTCTAAAATCATAATCAAAACAAATCTGGATTATATCAAATGGGATATG 903

60CSF_1680bp TTTGATTCTATTTCTAAAATCATAATCAAAACAAATCTGGATTATATCAAATGGGATATG 903

4559_2217bp TTTGATTCTATTTCTAAAATCATAATCAAAACAAATCTGGATTATATCAAATGGGATATG 1440

60B_594bp ---------------------------------------------------ATGGAATTT 9

60B_967bp AATCGTCATATAACAGATATTTATAGTATTGAACTTGATTCTGAACAGCAGATGGAATTT 963

60CSF_1680bp AATCGTCATATAACAGATATTTATAGTATTGAACTTGATTCTGAACAGCAGATGGAATTT 963

4559_2217bp AATCGTCATATAACAGATATTTATAGTATTGAACTTGATTCTGAACAGCAGATGGAATTT 1500

*********

60B_594bp GGTCATCGATATATCTTAGGTCTTTATCAGTTATTAGATCGTTTAATAACTAAGTTCCCT 69

60B_967bp GGTC-------------------------------------------------------- 967

60CSF_1680bp GGTCATCGATATATCTTAGGTCTTTATCAGTTATTAGATCGTTTAATAACTAAGTTCCCT 1023

4559_2217bp GGTCATCGATATATCTTAGGTCTTTATCAGTTATTAGATCGTTTAATAACTAAGTTCCCT 1560

****

60B_594bp TCAGTTCTATTTGAATCTTGTTCTTCAGGTGGTGGACGTTTTGATTTAGGACTTATGTAT 129

60B_967bp ------------------------------------------------------------ 967

60CSF_1680bp TCAGTTCTATTTGAATCTTGTTCTTCAGGTGGTGGACGTTTTGATTTAGGACTTATGTAT 1083

4559_2217bp TCAGTTCTATTTGAATCTTGTTCTTCAGGTGGTGGACGTTTTGATTTAGGACTTATGTAT 1620

60B_594bp TATGCACCGCAAGCGTGGACGAGTGATGATACGGACCCGATAGAAAGATTGAAAATTCAG 189

60B_967bp ------------------------------------------------------------ 967

60CSF_1680bp TATGCACCGCAAGCGTGGACGAGTGATGATACGGACCCGATAGAAAGATTGAAAATTCAG 1143

4559_2217bp TATGCACCGCAAGCGTGGACGAGTGATGATACGGACCCGATAGAAAGATTGAAAATTCAG 1680

60B_594bp CATGGAACTTCTTATGGATATTCTCCATCAATGATGACAGCCCATGTTTCTATTTCTCCA 249

60B_967bp ------------------------------------------------------------ 967

60CSF_1680bp CATGGAACTTCTTATGGATATTCTCCATCAATGATGACAGCCCATGTTTCTATTTCTCCA 1203

4559_2217bp CATGGAACTTCTTATGGATATTCTCCATCAATGATGACAGCCCATGTTTCTATTTCTCCA 1740

60B_594bp AATGAACAAAGTGGAAGACAAACGAGTTTGGACACTAGGACAAATGTAGCTTATTTTAGT 309

60B_967bp ------------------------------------------------------------ 967

60CSF_1680bp AATGAACAAAGTGGAAGACAAACGAGTTTGGACACTAGGACAAATGTAGCTTATTTTAGT 1263

4559_2217bp AATGAACAAAGTGGAAGACAAACGAGTTTGGACACTAGGACAAATGTAGCTTATTTTAGT 1800

60B_594bp TCTTTCGGTTATGAATTAGATGTTACGAGATTGTCGGTAGAAGAAAAAGAACAAGTGAGA 369

60B_967bp ------------------------------------------------------------ 967

60CSF_1680bp TCTTTCGGTTATGAATTAGATGTTACGAGATTGTCGGTAGAAGAAAAAGAACAAGTGAGA 1323

4559_2217bp TCTTTCGGTTATGAATTAGATGTTACGAGATTGTCGGTAGAAGAAAAAGAACAAGTGAGA 1860

60B_594bp GAACAAATTCAGTTTTATAAAAAATATCGTTCATTGCTTCAATATGGGGATTTCTATAGG 429

60B_967bp ------------------------------------------------------------ 967

60CSF_1680bp GAACAAATTCAGTTTTATAAAAAATATCGTTCATTGCTTCAATATGGGGATTTCTATAGG 1383

4559_2217bp GAACAAATTCAGTTTTATAAAAAATATCGTTCATTGCTTCAATATGGGGATTTCTATAGG 1920

60B_594bp ATAAACAGTCCTTTTAGTTGTGATTCTGCTAGTTGGCAAGTTGTTTCAAAAGATAAATGC 489

60B_967bp ------------------------------------------------------------ 967

60CSF_1680bp ATAAACAGTCCTTTTAGTTGTGATTCTGCTAGTTGGCAAGTTGTTTCAAAAGATAAATGC 1443

4559_2217bp ATAAACAGTCCTTTTAGTTGTGATTCTGCTAGTTGGCAAGTTGTTTCAAAAGATAAATGC 1980

60B_594bp CAATCGATTTTATTGTATGCTCAATTGAATAGTAAGTTGAATCCAGGTTATACAAGAGTT 549

60B_967bp ------------------------------------------------------------ 967

60CSF_1680bp CAATCGATTTTATTGTATGCTCAATTGAATAGTAAGTTGAATCCAGGTTATACAAGAGTT 1503

4559_2217bp CAATCGATTTTATTGTATGCTCAATTGAATAGTAAGTTGAATCCAGGTTATACAAGAGTT 2040

60B_594bp TATTTTAGTGGTTTAGATAAAGATCTGTCTCTTATACACATCTGA--------------- 594

60B_967bp ------------------------------------------------------------ 967

60CSF_1680bp TATTTTAGTGGTTTAGATAAAGATAAATGCTATTCCGTCTCTGGATTTGATGAGTTCTTT 1563

4559_2217bp TATTTTAGTGGTTTAGATAAAGATAAATGCTATTCCGTCTCTGGATTTGATGAGTTCTTT 2100

60B_594bp ------------------------------------------------------------ 594

60B_967bp ------------------------------------------------------------ 967

60CSF_1680bp TATGGAGATGAATTAATGAATGCTGGAATAAAAGTAAGTTTAAGTAATTTAGCACTTTGT 1623

4559_2217bp TATGGAGATGAATTAATGAATGCTGGAATAAAAGTAAGTTTAAGTAATTTAGCACTTTGT 2160

60B_594bp --------------------------------------------------------- 594

60B_967bp --------------------------------------------------------- 967

60CSF_1680bp GTTCCAGAATATCTTACAAAATTATTTGTTATTGAAGAAGTTGTATGTAAATATTGA 1680

4559_2217bp GTTCCAGAATATCTTACAAAATTATTTGTTATTGAAGAAGTTGTATGTAAATATTGA 2217
